# Determination of Flavonoid Glycoside Isomers Using Vision Transformer and Tandem Mass Spectrometry

**DOI:** 10.3390/plants13233401

**Published:** 2024-12-04

**Authors:** Ji In Park, Myeong Ji Kim, Kyu Hyeong Lee, Seung Hyun Oh, Young Hoon Kang, Hyunwoo Kim

**Affiliations:** College of Pharmacy and Integrated Research Institute for Drug Development, Dongguk University-Seoul, Goyang 10326, Republic of Korea; bajiin0624@dgu.ac.kr (J.I.P.); mjk980302@dgu.ac.kr (M.J.K.); asdasd4514@dgu.ac.kr (K.H.L.); dhtmdgus8121@gmail.com (S.H.O.); yhkang@dgu.ac.kr (Y.H.K.)

**Keywords:** flavonoid, artificial intelligence, vision transformer

## Abstract

A vision transformer (ViT)-based deep neural network was applied to classify the flavonoid glycoside isomers by analyzing electrospray ionization tandem mass spectrometry (ESI-MS/MS) spectra. Our model successfully classified the flavonoid isomers with various substitution patterns (3-O, 6-C, 7-O, 8-C, 4′-O) and multiple glycosides, achieving over 80% accuracy during training. In addition, the experimental spectra from flavonoid glycoside standards were acquired with different adducts, and our model showed robust performance regardless of the experimental conditions. As a result, the vision transformer-based computer vision model is promising for analyzing mass spectrometry data.

## 1. Introduction

Flavonoids, widely found in fruits and vegetables, are important constituents of the human diet and have been extensively researched for their potential health benefits, including anti-inflammatory effects, cancer prevention, cardiac protection, and antioxidative properties [1,2]. These compounds are structurally characterized by the arrangement of phenolic groups around a 15-carbon skeleton, which is often substituted by the attachment of sugar moieties in the case of flavonoid glycosides. This glycosylation can significantly influence the solubility, stability, and bioavailability of flavonoids, thereby affecting their biological activity and metabolism within the human body [3].

The position of glycosylation in flavonoid glycosides is typically determined using mass spectrometry (MS) and nuclear magnetic resonance (NMR). However, this analytical method has its limitations. In particular, MS analysis faces challenges in determining positional isomers due to their similar mass spectra and fragmentation patterns [4]. Positional isomers have the same molecular formula but differ only in the position of a functional group or substituent on the main scaffold, making it difficult to distinguish them based on molecular weights alone. Although positional isomers might produce similar mass spectra, subtle differences in their fragmentation patterns can sometimes be used for differentiation [5]. Interpreting these differences, however, can be challenging. For example, the location of a functional group in different positions can lead to varying fragment stability, but predicting or interpreting these differences requires detailed knowledge of ion chemistry and often advanced computational modeling.

To address this complexity and extract meaningful information from MS spectra, various computational approaches have been applied. The Global Natural Products Social (GNPS) Molecular Networking tool provides network analysis by measuring the similarity between MS2 fragmentation patterns to identify metabolites and offer similarity-based network analysis [6]. The SIRIUS platform, which includes the SIRIUS, CSI:FingerID, ZODIAC, and CANOPUS modules, analyzes isotopic and fragmentation patterns using a fragmentation tree algorithm and a support vector machine (SVM)-based multiple kernel learning method [7,8,9]. This platform enables the identification of molecular formulas and the structure of molecules and the prediction of molecular classes. MSNovelist is another tool used for the de novo structure elucidation of MS2 spectra [10].

Deep neural networks (DNNs) offer various advantages when analyzing highly complex data such as images, text, audio, and video. One of the most significant benefits of DNNs is their ability to automatically detect and learn relevant features from the data without the need for manual feature extraction [11]. This capability is particularly valuable for unstructured data, which does not fit easily into traditional, structured databases and often contains complex patterns that are difficult for humans to recognize and encode manually. Additionally, DNNs can be adapted to a wide range of data types and tasks and are relatively robust against noise in data, allowing them to focus on the features that truly matter for the task [12]. These advantages make DNNs well suited for dealing with MS data, which contains complex information along with noise.

The elucidation of flavonoid glycosides using MS has been extensively studied in analytical chemistry, offering numerous studies contributing to the identification and differentiation of flavonoid glycosides. Early work by Waridel et al. demonstrated the capability of the collisionally activated dissociation (CAD) of protonated flavonoid-C-glycosides to distinguish between 6-C and 8-C glycosides, setting a precedent for the differentiation of isomers [13]. After that, various studies introduced methodologies for characterizing O-diglycosyl and C-diglycosyl flavonoids through tandem MS, focusing on the abundance of fragmentation ions and the previously established rules of fragmentation patterns using MS2 or even MS3 [14,15,16]. This analysis provided practical guidelines for the identification of flavonoid isomers in complex crude mixtures. Hvattum et al. focused on the relative abundance between a deprotonated aglycone radical ion ((Y_0_–H)^−^) and aglycone radical ion (Y_0_^−^) to distinguish the sugar position of various flavonoid glycosides [17]. Additionally, Cuyckens et al. extended this radical ion-based approach from deprotonated to sodiated molecules [18]. Pikulski et al. further advanced the field by applying electrospray ionization tandem mass spectrometry (ESI-MS/MS) to explore the unique fragmentation patterns of metal/flavonoid complexes, which provided better sensitivity and more distinctive fragmentation patterns than observed for conventional protonated or deprotonated flavonoids [19]. The exploration of systemic approaches to tandem MS spectra continued with studies such as Ablajan et al.’s work on flavonoid O-rhamnosides, where negative ESI-MSn was utilized to differentiate isomeric compounds effectively through the comparison of the abundance of fragmentation ions as well as their diagnostic ions [20]. In more recent developments, Zhong et al. integrated a mass fragmentation and ion filtering strategy to accurately annotate 29 glycosylated flavones in green tea, highlighting the ongoing innovation in MS techniques for complex natural products analysis [21]. These studies have demonstrated that the glycosylation pattern in flavonoid glycosides influences the tandem MS spectra. Building on this understanding, we hypothesized that the automated identification of glycosylation patterns could be achieved through the application of computer vision technology that is especially designed to extract features from images.

In this study, a vision transformer (ViT), a state-of-the-art computer vision architecture, was modified for MS2 spectra analysis to differentiate flavonoid glycosides for the first time [22]. A ViT is a model that applies the principles of transformers, originally designed for natural language processing (NLP), to computer vision tasks [23]. In this architecture, images are divided into fixed-size patches, with each patch treated as the equivalent of a “word” in a language model. The key innovation of the ViT is its demonstration that transformers can effectively handle not just text but also images, without any convolutional layers, provided that they are trained on large enough datasets or with sufficient augmentation strategies.

## 2. Results 

### 2.1. Development of ViT Model for MS/MS Spectra

**Figure 1:**
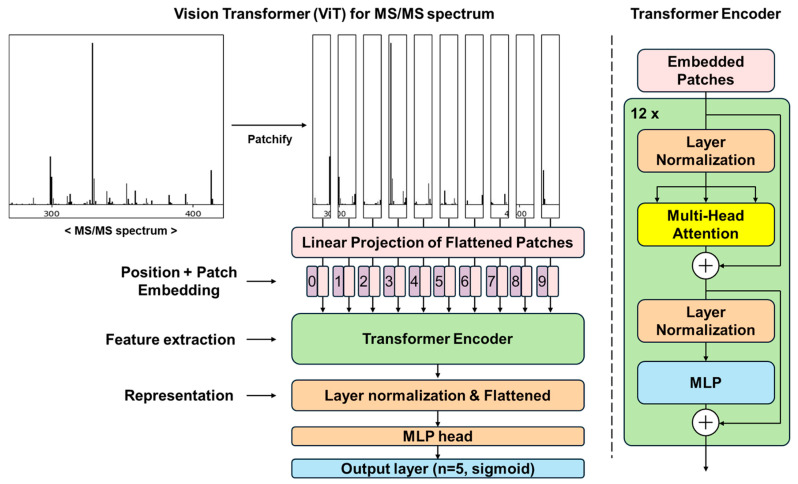
The ViT architecture for MS/MS spectra involves patchifying, flattening, and submitting the MS/MS spectra to a transformer encoder along with positional embeddings. The transformer encoder comprises 12 transformer blocks, each consisting of multi-head attention layers and multi-layer perceptrons. The extracted features from the encoder are then transferred to the MLP head, which predicts the glycosylation patterns in the output layers.

The architecture of the ViT model is described in Figure 1. The ViT model applies the transformer architecture with self-attention to sequences of image patches, without using convolution layers that are widely used in computer vision or image classification. In this model, MS/MS spectra are split into patches, through a process called “patchification”. However, since each patch lacks positional information, positional embeddings describing the location or position of an entity in a sequence are encoded and added to the patches. The encoded patches are then passed to twelve transformer encoder blocks, each consisting of a multi-head attention layer, a normalization layer, and a multi-layer perceptron (MLP) layer. All outputs from the transformer blocks are normalized, flattened, and then submitted to the MLP head for glycosylation pattern prediction. The activation function of the output layer is set to sigmoid to account for the multiple positions of sugar units.

### 2.2. Evaluation of the ViT Model on the Differentiation of Flavonoid Glycosides 

To evaluate the performance of the ViT model in predicting glycosylated patterns of flavonoid glycosides, a test set (n = 1745) of randomly chosen MS/MS spectra that were never used in the model training was prepared. Our model successfully identified flavonoid glycosides with an accuracy of 84.7% for the test set. However, accuracy alone does not fully demonstrate the model’s performance for each glycosylated pattern. Therefore, the precision, recall, and F1 score for each glycosylated pattern are detailed in Table 1. 

**Figure 2:**
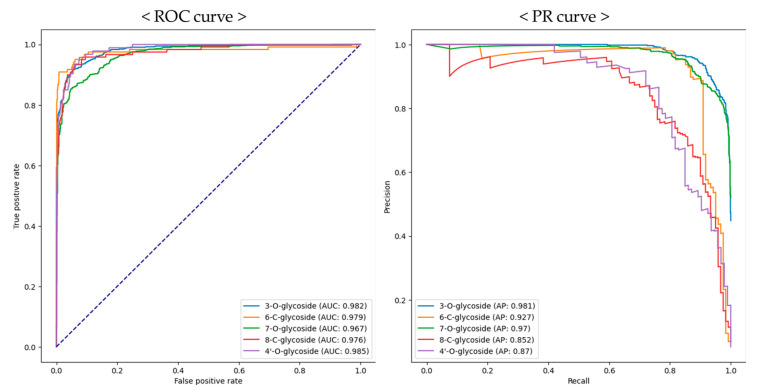
Receiver operating characteristic (ROC) curve and precision–recall curve for ViT model.

Our model demonstrated a good average precision (AP) of 0.818, recall of 0.874, and F1 score of 0.839 on the test set. The 8-C-glycoside category showed a lower precision (0.600) compared to other categories but exhibited a high recall (0.900). The receiver operating characteristic (ROC) curve, representing the sensitivity and specificity of the model, showed high area under the curve (AUC) values over 0.95 for all glycosylated patterns (Figure 2). While the ROC-AUC is a good metric for assessing algorithm performance, it has limitations in cases of data imbalance, such as with the 6-C-glycoside, 7-O-glycoside, and 4′-O-glycoside categories in our study. Therefore, the precision–recall area under the curve (PR-AUC) was measured to evaluate the model’s performance more accurately. As shown in Figure 2, all categories displayed great PR-AUC values over 0.8. Consequently, our model achieved excellent performance across all categories.

**Figure 3:**
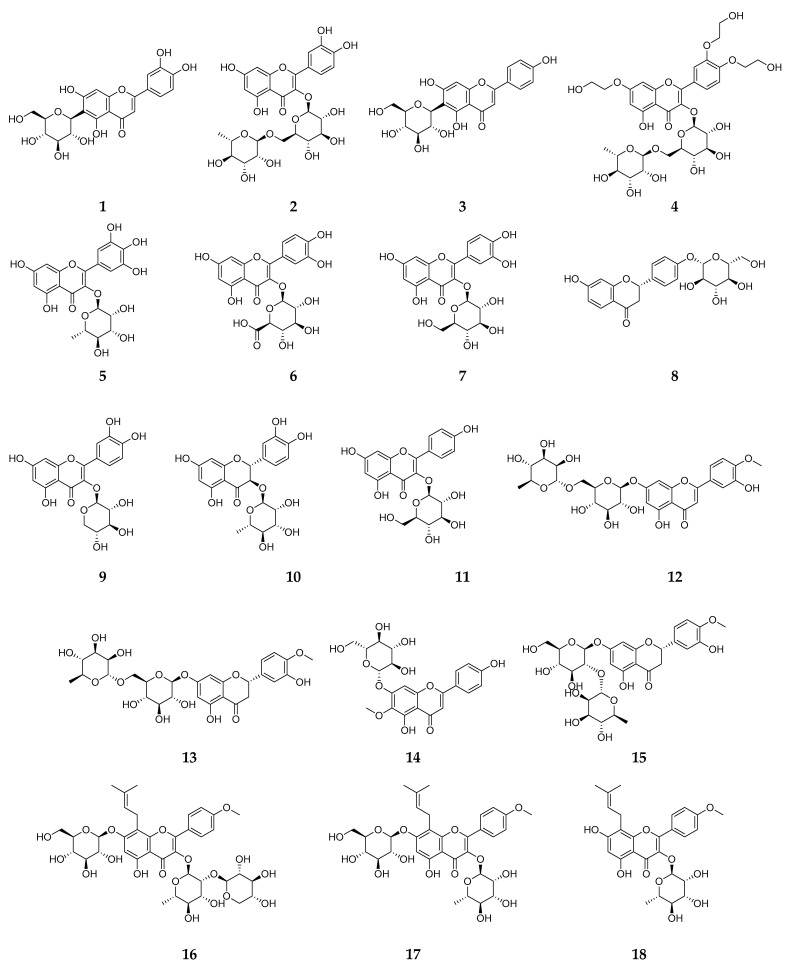
Structures of 18 flavonoid standards.

In machine learning, the discrepancy between training data and real-world data refers to differences between the data used for model training and the data encountered during real-world deployment. This discrepancy can affect the model’s performance and generalization ability. As shown in Table 2, 25 spectra (see Supplementary Materials S1) were obtained from 18 flavonoids (Figure 3), and most of the glycosylated patterns of the standards were correctly predicted, regardless of adduct type ([M+H]^+^ or [M+Na]^+^ were observed in our case).

### 2.3. Application of the ViT Model to Annotate Flavonoid Glycosides in the Citrus Unshiu Peel Extract

**Figure 4:**
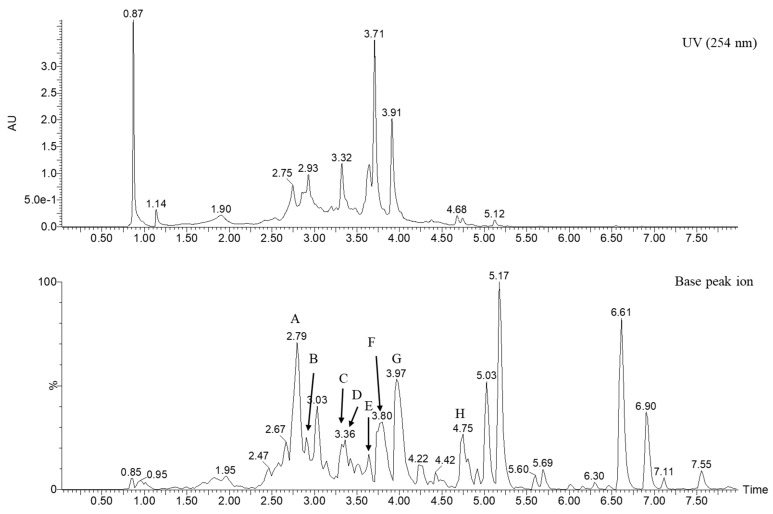
UV (254 nm) and base peak ion chromatogram of methanolic extract of *C. unshiu* peel.

The peel of *Citrus unshiu* is a source of flavonoid glycosides and is widely used in traditional Chinese medicine (TCM). For quality control and geographical discrimination, various studies have analyzed the contents of flavonoid glycosides in crude extracts. To identify or annotate flavonoid glycosides in the extract of *C. unshiu* peel, the ion chromatogram of the extract (Figure 4) was analyzed by GNPS to match the MS/MS fragmentation patterns with the spectrum library. As shown in Table 3, eight peaks (Peaks A–H; see Supplementary Materials S2) of flavonoid glycosides were detected in the ion chromatogram of the crude extract. Among them, six peaks (Peaks A, C, D, F, G, and H) were identified through a comparison of MS/MS fragmentation pattern libraries in GNPS (Task ID: e33f00ca8153486fb72d25016cc878c6; see Supplementary Materials S3).

**Figure 5:**
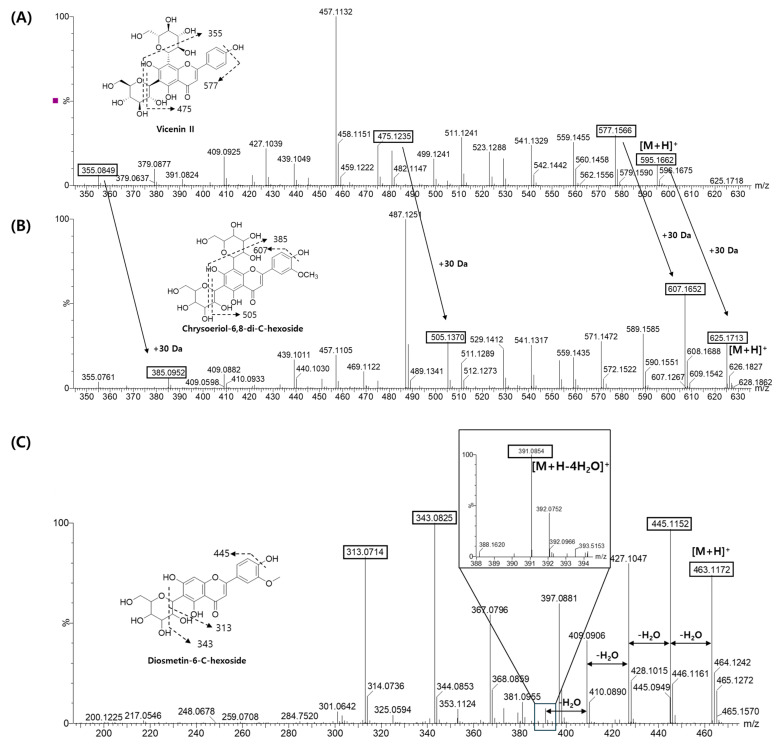
MS/MS fragmentations of Peak A (**A**), Peak B (**B**), and Peak E (**C**) at 20 eV of collision energy.

However, other peaks (Peaks B and E) were not directly identified by retrieving the MS spectrum library. Therefore, the molecular structures and glycosylation patterns of these peaks were manually annotated by analyzing their fragmentation patterns, including neutral losses. Peak B showed a molecular ion at *m*/*z* 625.1764 [M+H]^+^ (calcd for C_28_H_32_O_16_; error: 1.0 ppm), which is consistent with the molecular formula C_28_H_32_O_16_. The MS/MS spectra of peak B were similar to those of peak A (vicenin II), but most fragment ions of peak B were shifted by 30 Da, corresponding to the mass difference between peak A (595.1661) and peak B (625.1743). This shift indicated the presence of a methoxy group (-OCH_3_, 30 Da) on vicenin II (Figure 5A,B). In particular, the fragment ions at *m*/*z* 475 [M+H-120]^+^ and 385 [M+H-240]^+^ indicated the presence of a C-di-hexose moiety (Figure 5B). Consequently, peak B was annotated as chrysoeriol-6,8-di-C-hexoside, although the predicted result (7-O) from the ViT model was incorrect.

Peak E displayed a molecular ion at *m*/*z* 463.1232 [M+H]^+^ (calcd for C_22_H_22_O_11_; error: 1.7 ppm), which revealed the molecular formula C_22_H_22_O_11_. The fragment ions at *m*/*z* 343 [M+H-120]^+^ suggested the presence of a C-hexose moiety (Figure 5C). Flavonoid-mono-C-glycosides are generally substituted at the 6-C or 8-C positions, and these can be distinguished through the detection of a diagnostic product ion [M+H-4H_2_O]^+^, which is only observed in 6-C glycosides [24]. Thus, the fragment ion at *m*/*z* 391 [M+H-4H_2_O]^+^ confirmed that Peak E originated from a 6-C glycoside, which is in agreement with the prediction result (6-C). Diosmetin-6-C-glucose, which has the molecular formula C_22_H_22_O_11,_ has been reported in *C. unshiu* [25], so Peak E might be diosmetin-6-C-glucoside or its isomer.

**Table 3:** Flavonoid glycoside peaks in the LC-MS/MS profile of *C. unshiu* peel extract along with identified results.

Peak	RT	Formula	Adduct	Observed *m*/*z*	Annotation Results	Predicted Patterns
A	2.79	C_27_H_30_O_15_	[M+H]^+^	595.1661	Vicenin II ^a^ (6-C, 8-C)	6-C, 8-C
B	2.90	C_28_H_32_O_16_	[M+H]^+^	625.1763	Chrysoeriol-6,8-di-C-hexoside (6-C, 8-C)	7-O ^b^
C	3.36	C_26_H_28_O_14_	[M+H]^+^	565.1552	Isovitexin 2″-O-arabinoside^a^ (6-C)	6-C
D	3.42	C_27_H_30_O_15_	[M+H]^+^	595.1650	Nicotiflorine ^a^ (7-O)	7-O
E	3.64	C_22_H_22_O_11_	[M+H]^+^	463.1232	Diosmetin 6-C-glucoside/isomer [25] (6-C)	6-C
F	3.80	C_27_H_32_O_14_	[M+H]^+^	581.1871	Naringin ^a^ [26] (7-O)	7-O
G	3.97	C_28_H_34_O_15_	[M+H]^+^	611.1970	Hesperidin ^a^ [26] (7-O)	7-O
H	4.75	C_28_H_34_O_14_	[M+H]^+^	595.2010	Poncirin ^a^ [26] (7-O)	7-O

(^a^) Annotated results from GNPS library matching. (^b^) Incorrectly predicted result.

Interestingly, the predicted glycosylated patterns of those peaks were identical to those of the identified results without Peak B. These results demonstrate that the ViT model can be applied for the determination of flavonoids in natural products.

## 3. Discussion

In this study, we applied the vision transformer (ViT) architecture to analyze mass spectra, aiming for artificial intelligence to extract meaningful information from the spectra for differentiating flavonoid glycosides. As shown in Table 1, the ViT model successfully discriminated the major glycosylation patterns of flavonoid glycosides from their tandem mass spectra. The model’s performance was consistent with the experimental data from both standard compounds and the crude extract of *C. unshiu*. Additionally, adduct formations, such as protonation or sodiation, did not affect the prediction results (Table 2). These findings suggest that our approach can handle various adduct formations found in nature.

**Figure 6:**
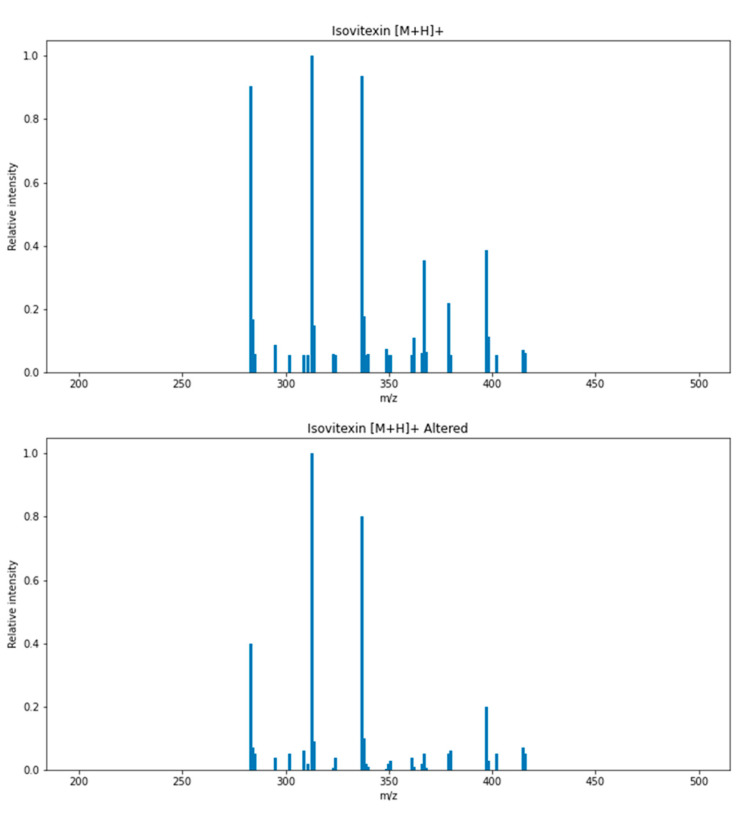
The MS/MS fragmentation pattern of isovitexin (3) and its altered fragmentation pattern, which was predicted as an 8-C-glycoside.

Generally, a deep learning method is referred to as a “black box” because it is difficult to understand how deep neural networks make their decisions. However, it is possible to infer how they work by making random changes to the data and observing how these changes affect the model’s predictions, a technique known as perturbation-based analysis. For this purpose, the intensities of fragmented ions of isovitexin (**3**) were randomly altered (Figure 6; see Supplementary Materials S4), which resulted in the prediction changing from 6-C-glycoside to 8-C-glycoside. This result demonstrates that the intensity ratio between fragment ions influenced the prediction, which is consistent with the findings of Pereira et al., who compared the fragmentation patterns between vitexin (8-C-glycoside) and isovitexin [27].

Ultimately, a deep learning approach is meaningful when the model can be applied to solve real-world challenges. The case study of *C. unshiu* peel extract demonstrates how our approach can be used to profile secondary metabolites in natural products. Table 3 illustrates how the fragment-based annotation results can be complemented by our model. Our model not only presented results consistent with the annotation results but also suggested glycosylation patterns for other flavonoid glycosides that had not been previously annotated.

Currently, there are no directly comparable tools available that offer the same functionality as our ViT-based model for predicting glycosylation patterns in flavonoid glycosides using MS/MS data. While several tools exist for general metabolite identification, they do not focus specifically on the detailed glycosylation pattern recognition that our model aims to achieve. As such, a direct comparison with existing tools could not be performed in this study.

## 4. Materials and Methods

### 4.1. General Experimental Procedures

All flavonoid glycoside standards for the experimental evaluation were purchased from MedChem Express Co. Ltd. (Trenton, NJ, USA). Tandem MS spectra for the 18 flavonoid glycosides were acquired using a Waters Xevo G2 QTOF mass spectrometer (Waters Co., Manchester, UK) equipped with a Waters Acquity UPLC system (Waters Co., Milford, MA, USA) including a binary solvent delivery system, autosampler, and photodiode array (PDA) detector. The UPLC column was the Waters Acquity UPLC BEH C18 (150 mm × 2.1 mm, 1.7 μm). All solvents and reagents were purchased from Daejung Chemicals & Metals Co. Ltd. (Si-Heung, Republic of Korea).

### 4.2. Dataset Preparation

The MS/MS spectra for the training dataset were established from a GNPS library, where 499,450 MS/MS spectra were curated, including chemical structures, precursor ions, and adducts. Ion intensities of the MS/MS spectra were normalized, and spectra with fewer than 2 ions were removed. Among the spectra, those of flavonoid glycosides were filtered using NPClassifier, an artificial intelligence designed specifically to classify natural products [28]. The glycosylation patterns of flavonoid glycosides were curated and labeled through manual processes. In detail, (1) the structures of the collected flavonoid glycosides were drawn, and (2) the presence of five common glycosylation types (3-O, 6-C, 7-O, 8-C, and 4′-O) was confirmed. Subsequently, (3) the glycosylation pattern was labeled in a binary format, with 1 indicating the presence and 0 indicating the absence of each type. These steps were double-checked to ensure accuracy. In total, 17,615 spectra were labeled and established as a dataset for training. This dataset was split in a stratified fashion based on the glycosylation patterns: 80% were assigned to the training set; 10%, to the validation set; and 10%, to the test set. After hyperparameter tuning, the training and validation sets were merged and subjected to the final model training.

### 4.3. Preparation of an External Evaluation Test Set

To evaluate and compare the performance between different platforms using real data, experimental MS/MS spectra were prepared. Eighteen flavonoid glycoside standards were dissolved in methanol at a concentration of 0.1 mM. For the case study, dried peels of *C. unshiu* (0.1 g) that were provided from Dongguk University Ilsan Oriental Hospital were ground and extracted with MeOH (10 mL) using ultrasonication (1 h) at room temperature, followed by centrifugation (14,000 rpm, 10 min). The supernatant was then subjected to the LC-MS/MS system equipped with a Waters Acquity UPLC BEH C_18_ (150 mm × 2.1 mm, 1.7 μm) column with a CH_3_CN/H_2_O gradient system (10:90 to 99:1) in 15 min. The flow rate was set at 300 μL/min, and the injection volume was 2.0 μL. The MS/MS spectra of the standards and extract were acquired on a Waters Xevo G2 QTOF mass spectrometer connected to the UPLC system through an electrospray ionization (ESI) interface. The ESI conditions were set as follows: positive ion mode, capillary voltage of 2.5 kV, cone voltage of 40 V, source temperature of 120 °C, desolvation gas temperature of 250 °C, cone gas flow of 0 L/h, and desolvation gas flow of 1000 L/h. The ion acquisition rate was 0.2 s, and the collision energy was set to 20 eV. Data were centroided during acquisition using an independent reference lock mass ion via the LockSpray^TM^ interface to ensure accuracy and precision. Leucine enkephalin (*m*/*z* 556.2771 in positive mode) was used at a concentration of 2 ng/μL with an infusion rate of 5 μL/min. The data processing of MS/MS spectra was performed using MZmine 3.9.0 software [29], utilizing the processing wizard with preset parameters for UPLC-QTOF-DDA (data-dependent acquisition). All MS/MS spectra were then exported in MGF (mascot generic format) files.

### 4.4. Model Training

The training of neural networks was performed on a workstation equipped with an AMD Ryzen™ 9 5950X CPU, two NVIDIA^®^ RTX A6000 GPUs with 48 GB of video memory each, and 128 GB of RAM. Python 3.9 programming was used for this project, and the TensorFlow 2.15.0 deep learning framework was utilized. The vision transformer (ViT) was modified and trained to handle MS/MS spectra. A hyperband strategy provided by Keras Tuner was applied to find the optimal hyperparameters. An early stopping method was used to avoid overfitting; specifically, training was terminated if it exceeded a patience level of 10 epochs without a significant decrease in loss. The dataset was divided into training, validation, and test sets, with the test set withheld from the training process. Model performance was evaluated using the accuracy, precision, recall, and F1 score for each glycosylated pattern on the test set.

## 5. Conclusions

In this study, we successfully applied a vision transformer (ViT) model to the analysis of MS/MS spectra, demonstrating its efficacy in distinguishing flavonoid glycosides based on their glycosylation patterns. The ViT model, adapted from its original use in computer vision tasks to handle complex MS/MS data, showed high accuracy in identifying glycosylation patterns across various flavonoid glycosides. Our model achieved an overall accuracy of 84.7% for the test set and 96.0% for experimental data, indicating its robustness and potential for practical applications.

Furthermore, the ViT model proved capable of handling different adduct formations without compromising prediction accuracy. The application of our model to *C. unshiu* peel extract showed its practical utility in profiling secondary metabolites, accurately predicting glycosylation patterns consistent with conventional annotation methods. Thus, our approach complements existing methods and offers a promising tool for the comprehensive analysis of complex natural products.

## Figures and Tables

**Table 1:** The precision, recall, and F1 score of the glycosylation prediction results (n = 1745).

Glycosylated Pattern	Precision	Recall	F1 Score
3-O-glycoside (n = 782)	0.941	0.904	0.922
6-C-glycoside (n = 121)	0.893	0.893	0.893
7-O-glycoside (n = 909)	0.946	0.856	0.899
8-C-glycoside (n = 120)	0.600	0.900	0.720
4′-O-glycoside (n = 93)	0.710	0.817	0.760
Average (n = 1745)	0.818	0.874	0.839

**Table 2:** The list of 18 flavonoid standards and the prediction results.

No.	Compound Name	Formula	Adduct	Observed *m*/*z*	True Patterns	Predicted Patterns
1	Isoorientin	C_21_H_20_O_11_	[M+H]^+^	449.1069	6-C	6-C
2	Rutin	C_27_H_30_O_16_	[M+H]^+^	611.1604	3-O	3-O
			[M+Na]^+^	633.1442	3-O	3-O
3	Isovitexin	C_21_H_20_O_10_	[M+H]^+^	433.112	6-C	6-C
			[M+Na]^+^	455.0928	6-C	6-C
4	Troxerutin	C_33_H_42_O_19_	[M+Na]^+^	765.2219	3-O	3-O
5	Myricitrin	C_21_H_20_O_12_	[M+Na]^+^	487.0807	3-O	3-O
6	Miquelianin	C_21_H_18_O_13_	[M+H]^+^	479.081	3-O	3-O
			[M+Na]^+^	501.0627	3-O	3-O
7	Isoquercitrin	C_21_H_20_O_12_	[M+H]^+^	465.1022	3-O	3-O
			[M+Na]^+^	487.084	3-O	3-O
8	Liquiritin	C_21_H_22_O_9_	[M+Na]^+^	441.1138	4′-O	4′-O
9	Reynoutrin	C_20_H_18_O_11_	[M+Na]^+^	457.0714	3-O	3-O
10	Astilbin	C_21_H_22_O_11_	[M+Na]^+^	473.1051	3-O	3-O
11	Astragalin	C_21_H_20_O_11_	[M+Na]^+^	471.0892	3-O	7-O *
12	Diosmin	C_28_H_32_O_15_	[M+H]^+^	609.1781	7-O	7-O
13	Hesperidin	C_28_H_34_O_15_	[M+H]^+^	611.1966	7-O	7-O
			[M+Na]^+^	633.1799	7-O	7-O
14	Homoplantaginin	C_22_H_22_O_11_	[M+Na]^+^	485.1048	7-O	7-O
15	Neohesperidin	C_28_H_34_O_15_	[M+H]^+^	611.1981	7-O	7-O
			[M+Na]^+^	633.1798	7-O	7-O
16	Epimedin B	C_38_H_48_O_19_	[M+H]^+^	809.2871	3-O, 7-O	3-O, 7-O
			[M+Na]^+^	831.2613	3-O, 7-O	3-O, 7-O
17	Icariin	C_33_H_40_O_15_	[M+H]^+^	677.2457	3-O, 7-O	3-O, 7-O
18	Baohuoside	C_27_H_30_O_10_	[M+Na]^+^	537.1725	3-O	3-O

* Incorrectly predicted results.

## Data Availability

The source code for this study is available on GitHub at https://github.com/ChungmaruQ/Vit_Flavonoid (accessed on 4 August 2024). The dataset and weights of the trained model are available at https://zenodo.org/records/13208825 (accessed on 4 August 2024).

## Supplementary Materials

The following supporting information can be downloaded at https://www.mdpi.com/article/10.3390/plants13233401/s1: Supplementary Materials S1: MS/MS spectra of 18 flavonoid standards at CE 20 eV. Supplementary Materials S2: MS/MS of Peaks A to J in *C. unshiu* at CE 20 eV. Supplementary Materials S3: Mirror plots between Peaks A to J in *C. unshiu* and annotated compounds from GNPS library. Supplementary Materials S4: Altered MS/MS spectra of isovitexin (3).
