# Supplementary material for: Determination of Flavonoid Glycoside Isomers Using Vision Transformer and Tandem Mass Spectrometry
Source: Plants (Basel). 2024 Dec 4;13(23):3401. doi: 10.3390/plants13233401 (PMC11644359; doi:10.3390/plants13233401)
Supplement: Supplementary file 1 [file plants-13-03401-s001.zip › plants-3253070-supplementary.pdf]

Supporting information

Determination of Flavonoid Glycoside Isomers Using Vision Transformer and Tandem Mass Spectrometry

Ji In Park, Myeong Ji Kim, Kyu Hyeong Lee, Seung Hyun Oh, Young Hoon Kang, and Hyunwoo Kim*

College of Pharmacy and Integrated Research Institute for Drug Development, Dongguk University-Seoul, Goyang, Republic of Korea

* Correspondence: hwkim8906@dongguk.edu; Tel.:+82-31-961-5224

Table of contents

S1. MS/MS spectra of 18 flavonoid standards at CE 20 eV

S2. MS/MS of Peaks A to J in *C. unshiu* at CE 20 eV

S3. Mirror plots between Peaks A to J in *C. unshiu* and annotated compounds from GNPS library

S4. Altered MS/MS spectra of isovitexin (3)

S1. MS/MS spectra of 18 flavonoid standards at CE 20 eV

1) Isoorientin [M+H]+

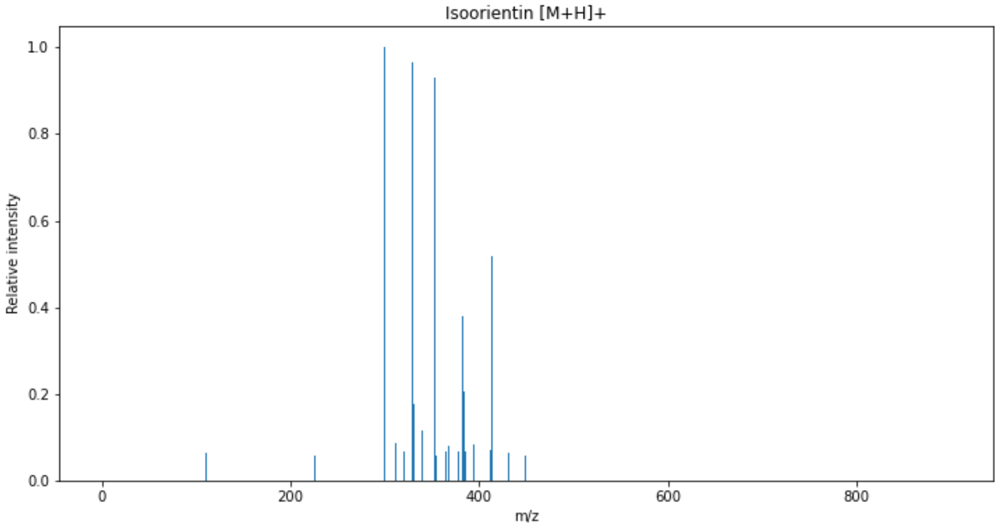

***m/z* (intensity):** 111.5132 (19), 226.8614 (17), 299.0361 (75), 299.0532 (290), 299.0677 (61), 300.0409 (24), 300.0534 (78), 311.0778 (25), 321.0777 (20), 329.0662 (280), 330.0494 (51), 330.058 (20), 331.0757 (35), 339.0877 (34), 353.064 (270), 354.0503 (17), 355.0465 (17), 365.0487 (20), 365.0718 (17), 367.0691 (23), 378.0626 (17), 378.0908 (20), 383.0687 (110), 383.0891 (18), 384.0441 (17), 384.0642 (17), 384.0821 (60), 385.0927 (20), 395.0258 (24), 395.0549 (17), 395.0877 (21), 412.6036 (21), 413.0761 (150), 413.0935 (56), 413.1526 (18), 414.0825 (41), 414.1063 (25), 431.0704 (19), 449.1149 (17)

2) Rutin [M+H]+

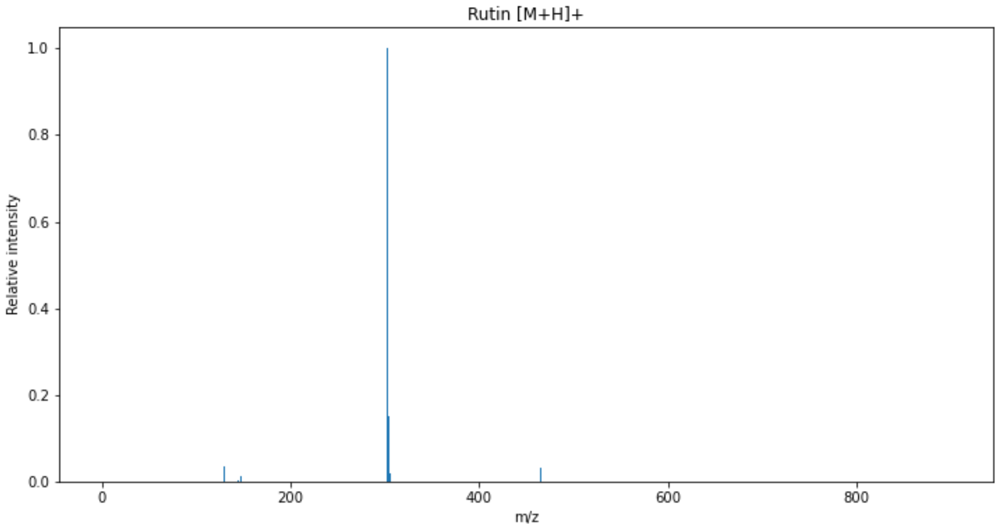

***m/z* (intensity):** 129.0458 (206), 129.0569 (1790), 145.0486 (170), 147.067 (654), 147.0733 (143), 148.0753 (102), 303.0069 (198), 303.052 (52400), 304.0289 (195), 304.0541 (7940), 305.061 (1090), 306.0599 (148), 465.0653 (260), 465.1075 (1750), 466.1073 (501)

3) Rutin [M+Na]+

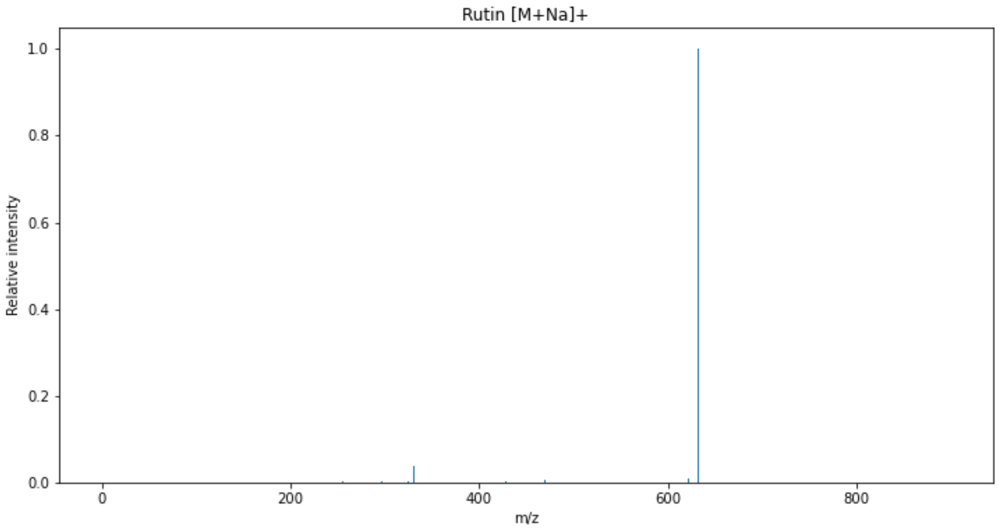

***m/z* (intensity):** 255.0828 (26), 296.6639 (29), 325.053 (25), 331.0997 (270), 429.0691 (23), 470.0799 (36), 622.6037 (57), 632.6786 (24), 633.1327 (6950), 633.1663 (1570), 633.2186 (38), 634.1518 (1940), 634.1829 (97), 635.1425 (430), 635.1782 (56), 636.135 (37), 636.1658 (87)

4) **Isovitexin [M+H]+**

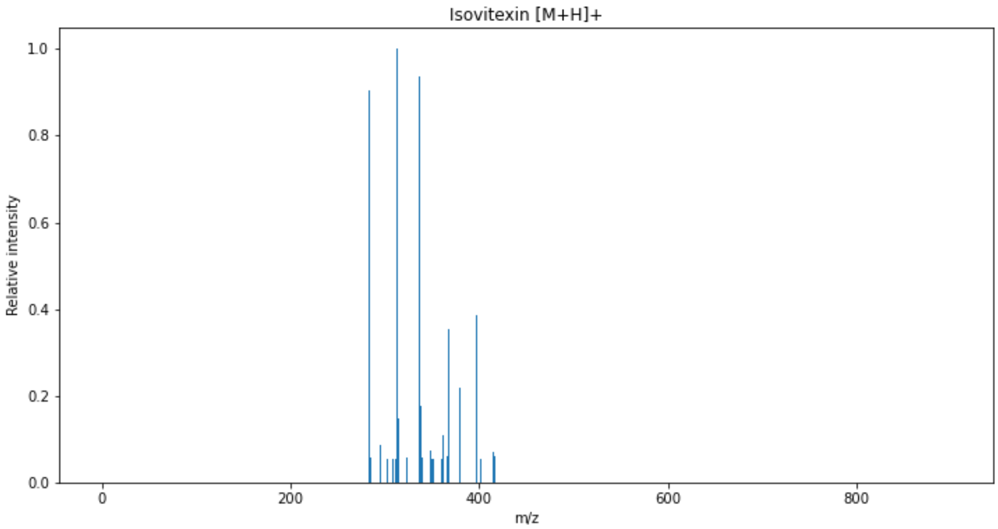

***m/z* (intensity):** 283.0467 (72), 283.0596 (280), 283.2373 (17), 284.0491 (50), 284.063 (52), 285.0671 (18), 295.0527 (19), 295.0668 (27), 302.0304 (17), 309.0574 (17), 311.0542 (17), 313.067 (310), 313.1053 (17), 314.0713 (46), 323.0942 (18), 324.0988 (17), 337.0297 (24), 337.0633 (290), 337.0798 (120), 338.0642 (55), 339.086 (17), 340.8889 (18), 349.0605 (23), 349.0791 (18), 350.0659 (17), 351.0837 (17), 361.0766 (17), 362.079 (34), 366.632 (19), 367.0737 (110), 367.0956 (19), 368.0805 (20), 379.0758 (68), 379.0952 (17), 380.0903 (17), 397.0717 (18), 397.0922 (120), 397.1192 (19), 398.0898 (35), 402.5657 (17), 415.0816 (17), 415.1033 (22), 415.1148 (17), 416.1013 (19)

5) **Isovitexin [M+ Na]+**

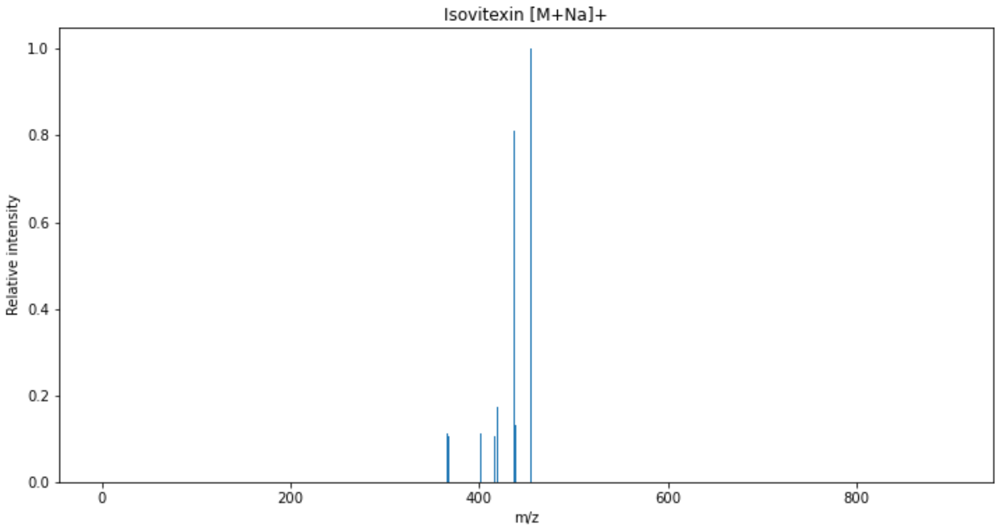

***m/z* (intensity):** 366.0519 (18), 367.072 (17), 402.0813 (18), 417.0395 (17), 419.1843 (28), 437.0563 (17), 437.0806 (130), 438.0833 (18), 438.1075 (21), 455.0682 (21), 455.0914 (160), 455.1166 (18), 456.1236 (18)

6) **Troxerutin [M+ Na]+**

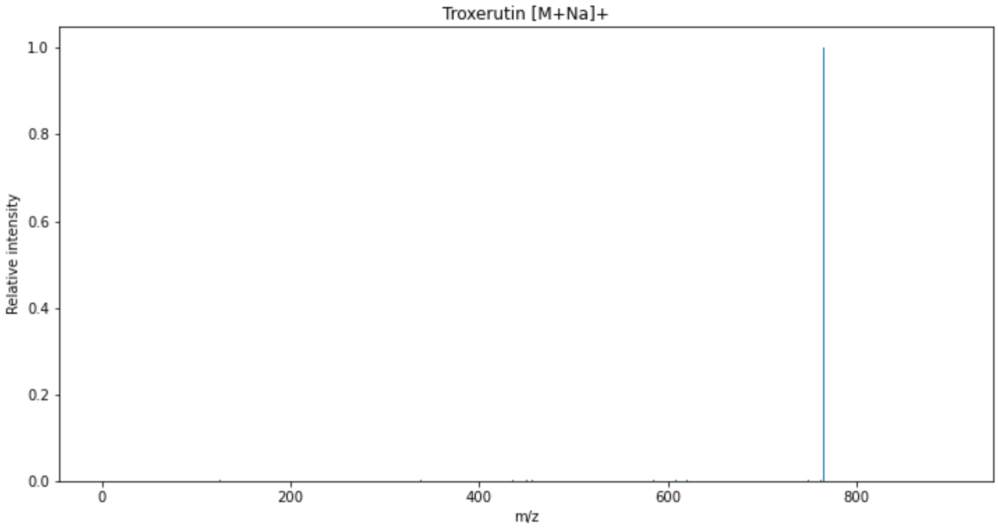

***m/z* (intensity):** 338.3368 (17), 436.1076 (17), 451.851 (21), 457.0916 (28), 457.1164 (18), 585.152 (21), 609.1677 (20), 621.1273 (19), 750.5187 (19), 762.0258 (20), 763.7305 (19), 765.1125 (21), 765.2221 (6700)

7) **Myricitrin [M+ Na]+**

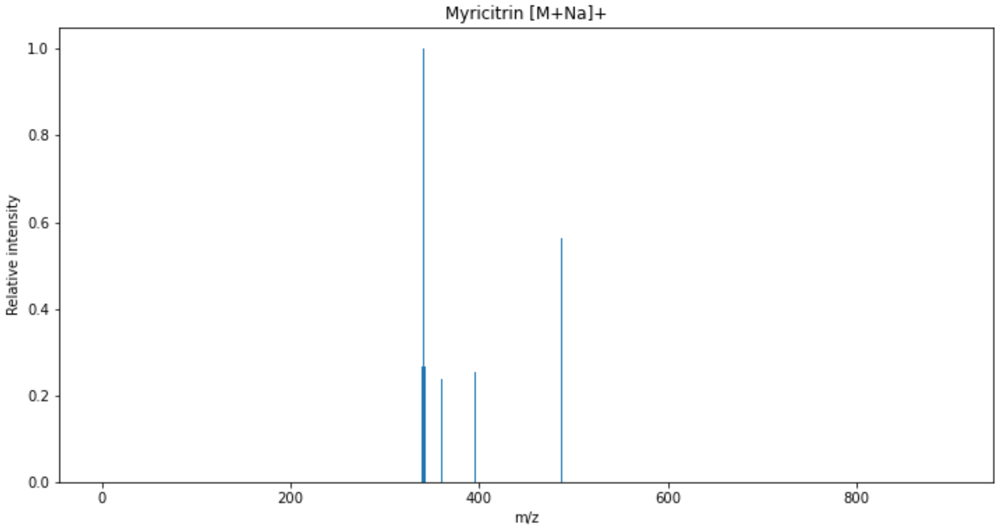

***m/z* (intensity):** 340.9918 (19), 341.0122 (71), 341.0257 (32), 343.0364 (19), 361.0102 (17), 396.6481 (18), 487.0818 (40), 487.1233 (21), 488.0742 (19), 488.0855 (38)

8) **Miquelianin [M+ H]+**

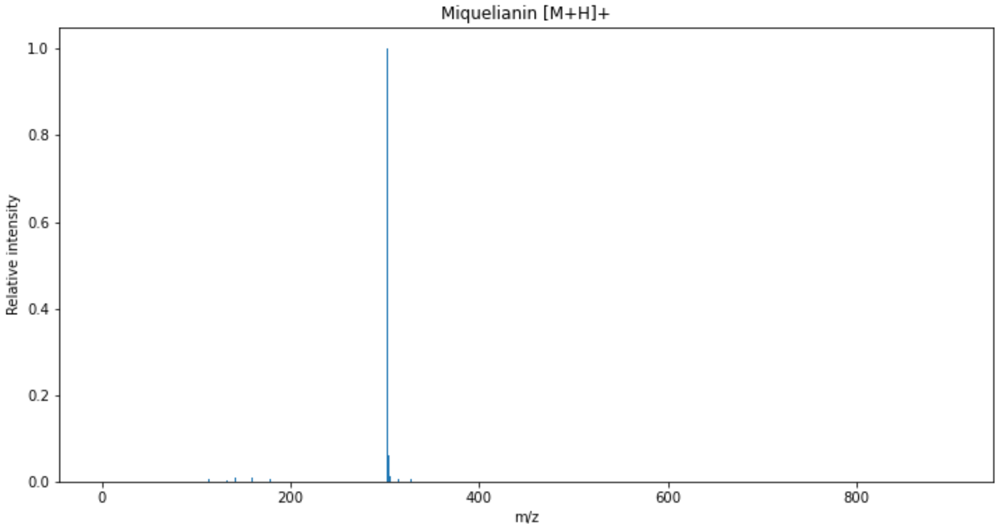

***m/z* (intensity):** 113.0186 (19), 132.0347 (17), 141.0208 (37), 159.0151 (17), 159.0275 (38), 179.582 (21), 302.0797 (19), 303.0494 (3700), 303.1239 (18), 304.0323 (51), 304.0476 (230), 304.062 (69), 305.0534 (42), 305.0668 (18), 305.2988 (18), 315.0575 (21), 328.0566 (18)

9) **Miquelianin [M+ Na]+**

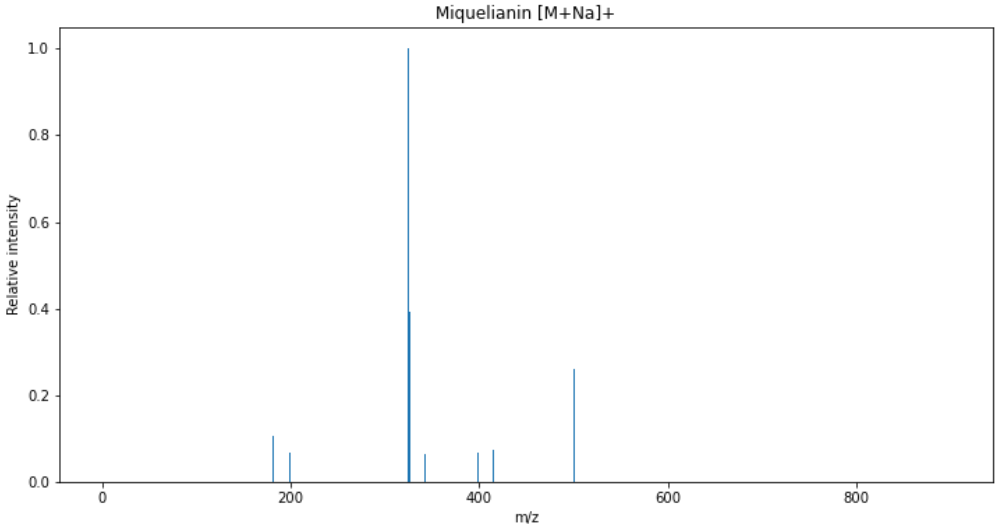

***m/z* (intensity):** 181.0188 (30), 199.0307 (19), 325.0147 (86), 325.0337 (280), 326.0274 (110), 327.0356 (43), 343.9917 (18), 399.0227 (19), 415.0028 (21), 501.0727 (73), 573.7662 (19)

10) **Isoquercitrin [M+ H]+**

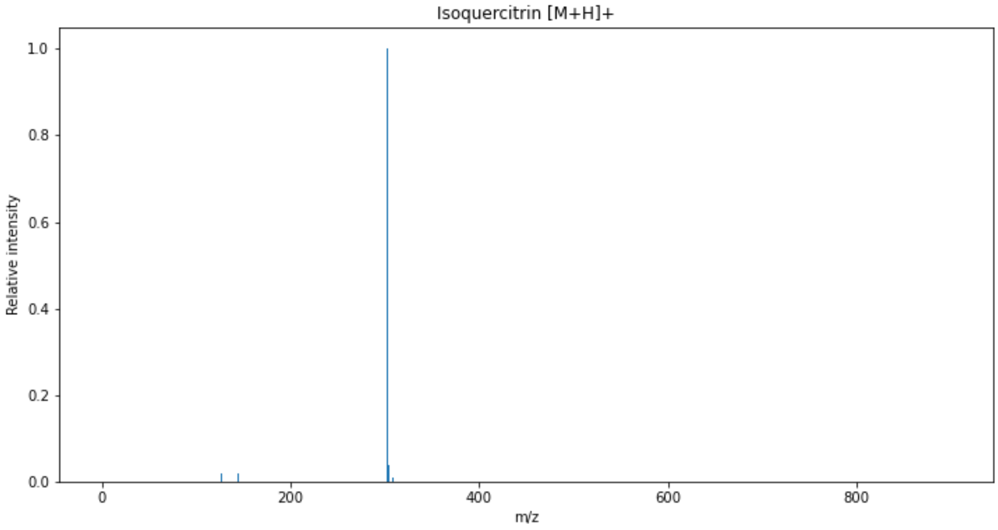

***m/z* (intensity):** 127.0304 (46), 127.0377 (29), 145.0503 (41), 302.0426 (17), 303.0485 (2200), 304.0341 (19), 304.0447 (83), 308.2551 (19)

11) **Isoquercitrin [M+ Na]+**

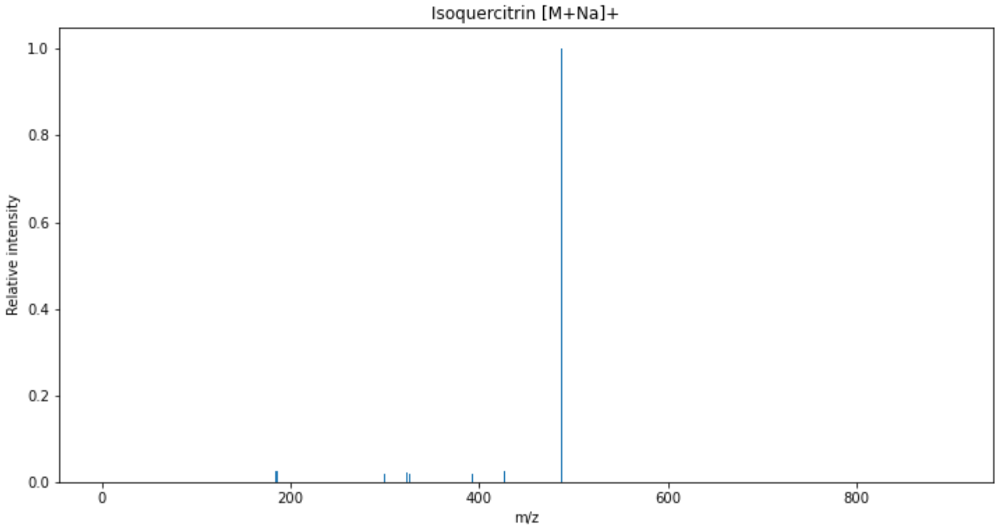

***m/z* (intensity):** 185.0436 (21), 300.3625 (17), 324.0302 (19), 326.0421 (17), 393.5365 (17), 427.1666 (22), 487.0803 (830), 487.1208 (17), 487.6897 (25), 488.0793 (130), 488.0975 (38), 489.0824 (20)

12) Liquirtin [M+ Na]+

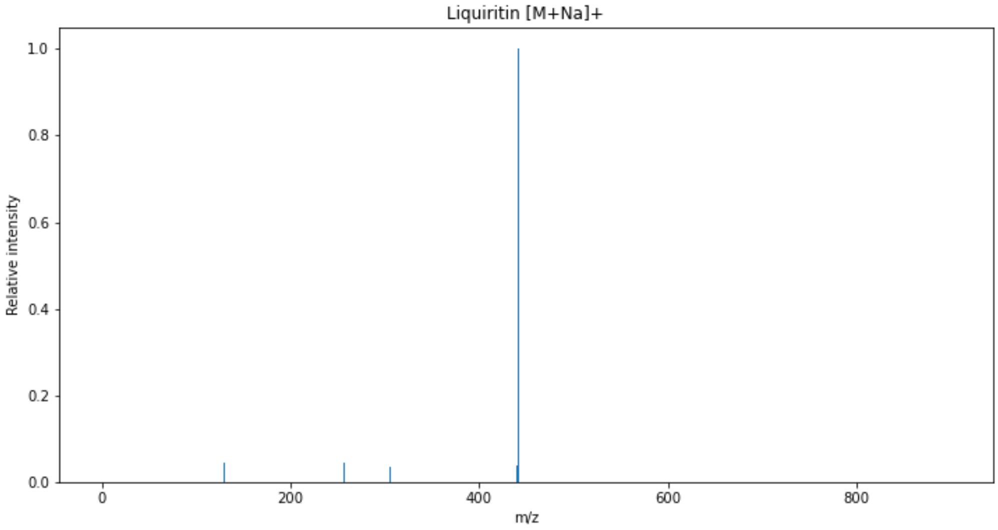

***m/z* (intensity):** 129.0173 (20), 257.0746 (20), 305.0898 (17), 440.6954 (18), 441.1004 (290), 441.1159 (460), 442.0838 (36), 442.105 (66), 442.1315 (17), 443.1 (34)

13) Reynoutrin [M+ Na]+

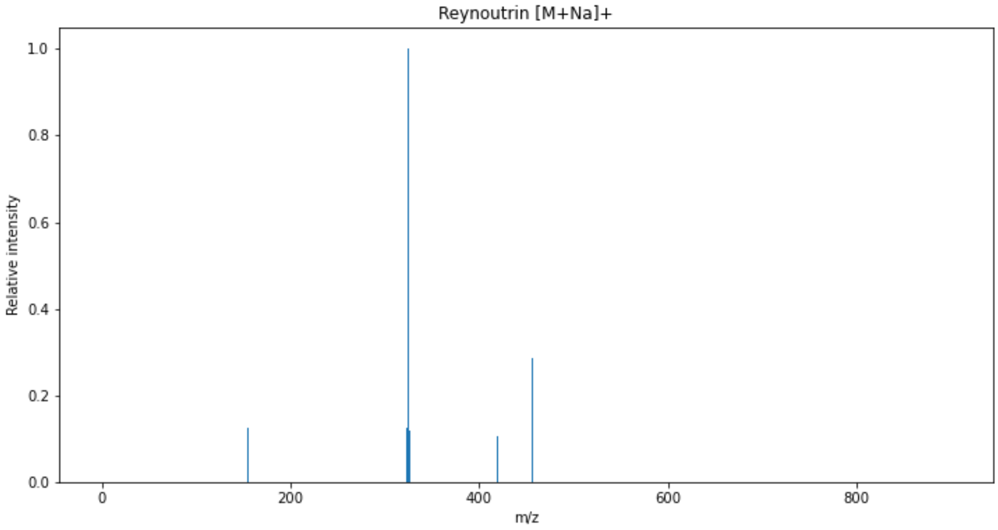

***m/z* (intensity):** 155.0239 (20), 324.0264 (20), 325.0285 (160), 325.0602 (18), 326.0196 (19), 326.0385 (17), 419.0112 (17), 457.0534 (46), 457.0695 (19), 457.0888 (18), 458.0714 (18)

14) Astilbin [M+ Na]+

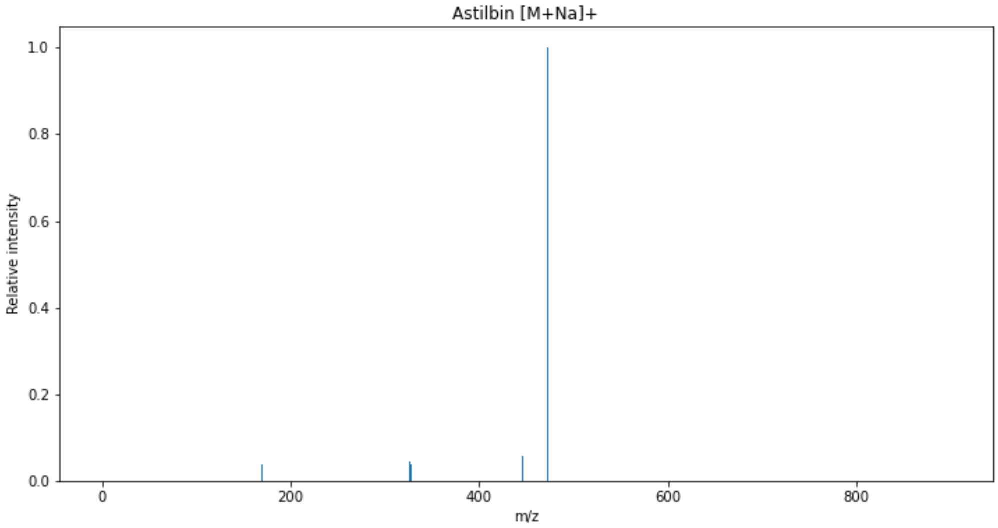

***m/z* (intensity):** 169.0424 (17), 326.6417 (17), 327.031 (20), 328.0445 (17), 446.0886 (25), 473.0979 (430)

15) Astragalin [M+ Na]+

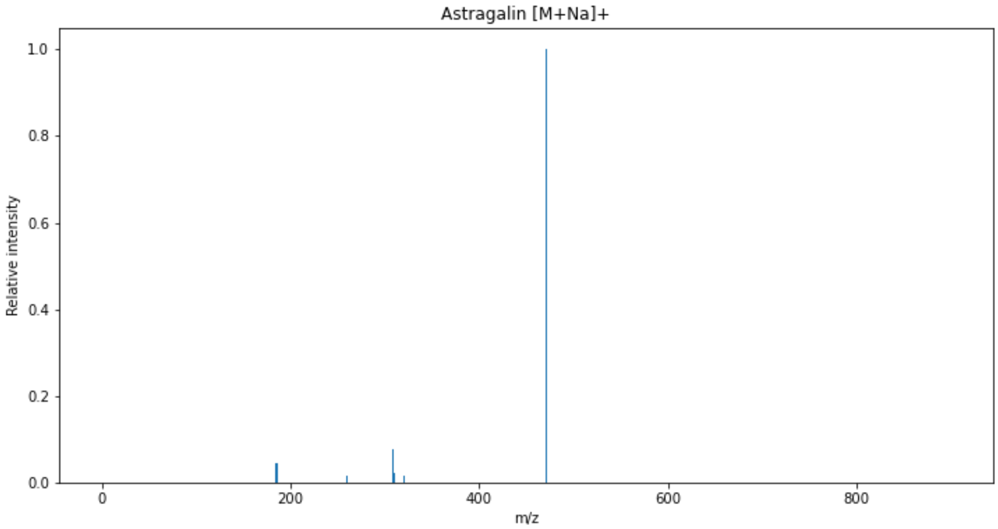

***m/z* (intensity):** 185.037 (54), 185.0481 (17), 186.0482 (17), 260.1114 (19), 309.0218 (93), 309.0356 (60), 309.0491 (17), 310.04 (28), 321.9895 (21), 471.0563 (19), 471.0949 (1200), 471.2118 (17), 472.057 (23), 472.0974 (140), 473.088 (17), 473.2073 (23), 474.0791 (17)

16) Diosmin [M+H]+

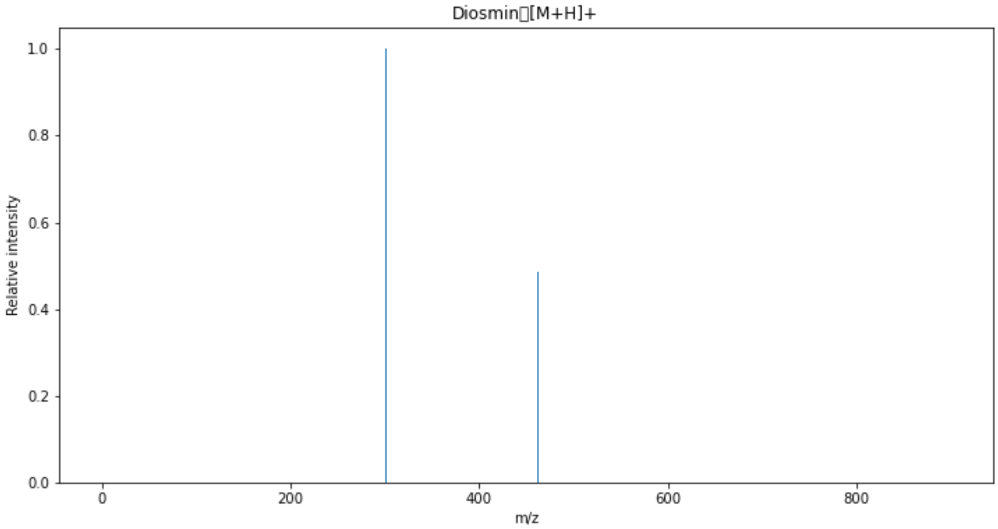

***m/z* (intensity):** 301.0627 (34), 301.08 (37), 462.6228 (17), 463.1247 (18)

17) Hesperidin [M+H]+

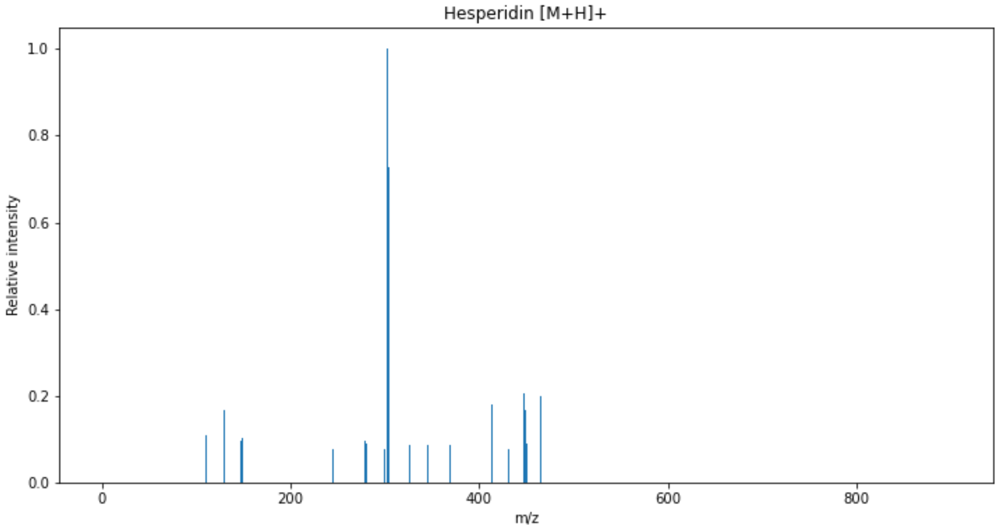

***m/z* (intensity):** 111.0376 (24), 129.0477 (37), 129.057 (17), 147.0738 (21), 149.0711 (23), 245.0516 (17), 279.0845 (21), 281.0637 (20), 299.0695 (17), 303.0731 (120), 303.0858 (220), 304.0841 (160), 327.0681 (19), 327.083 (19), 345.0728 (19), 345.0923 (17), 369.0801 (17), 369.1115 (19), 413.1377 (40), 414.1059 (20), 432.1407 (17), 448.1458 (45), 448.1559 (17), 449.1245 (19), 449.1423 (37), 450.1378 (20), 450.4381 (17), 465.1246 (44)

18) Hesperidin [M+Na]+

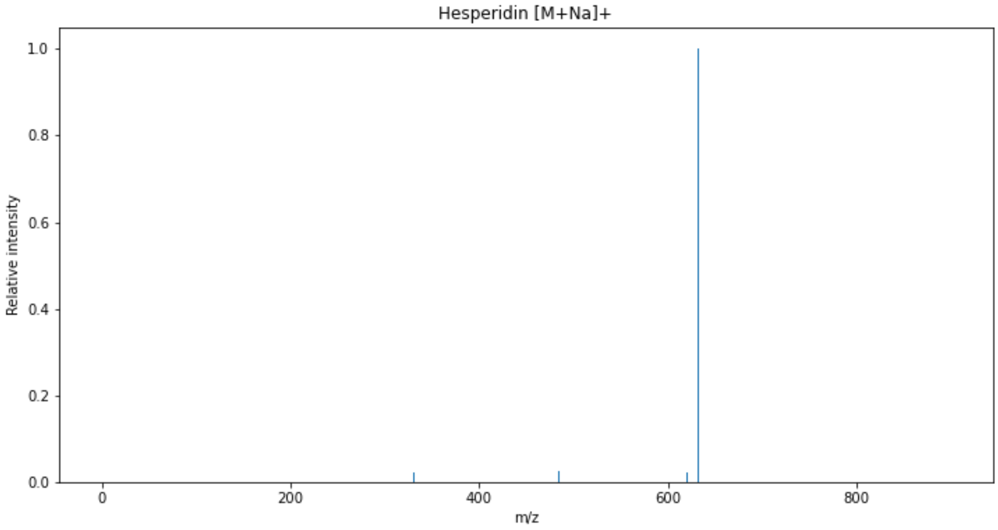

***m/z* (intensity):** 330.1574 (17), 331.0925 (18), 484.1016 (20), 621.0645 (17), 633.1041 (20), 633.1318 (36), 633.1606 (440), 633.1841 (750), 633.2209 (72), 634.1311 (24), 634.1732 (110), 634.2007 (76), 635.1702 (83), 635.1954 (17), 638.8502 (17)

19) Homoplantaginin [M+Na]+

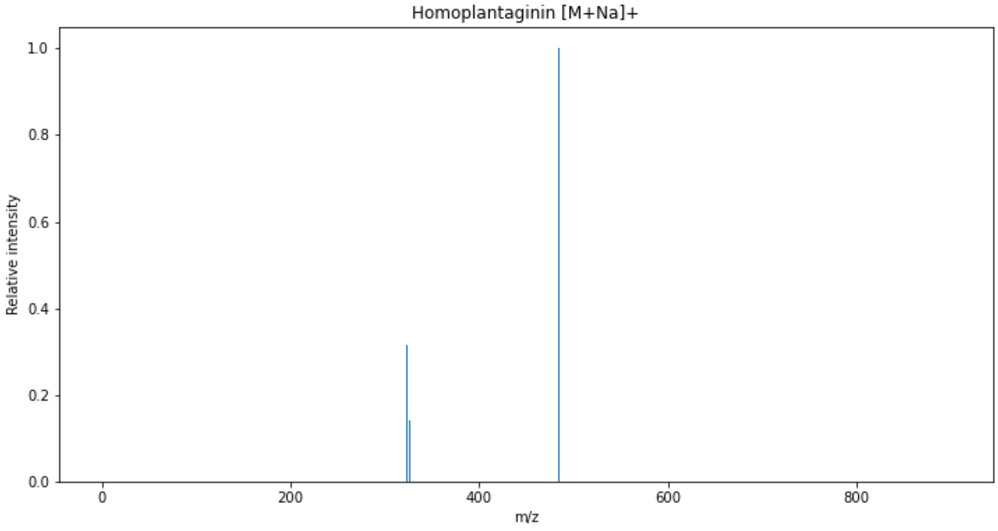

***m/z* (intensity):** 323.0486 (29), 323.061 (38), 324.0492 (17), 327.1951 (17), 485.0828 (78), 485.1028 (120), 485.1204 (36), 485.2108 (19), 486.0749 (25), 486.1145 (35), 487.0927 (26), 493.7724 (20)

20) Neohesperidin [M+H]+

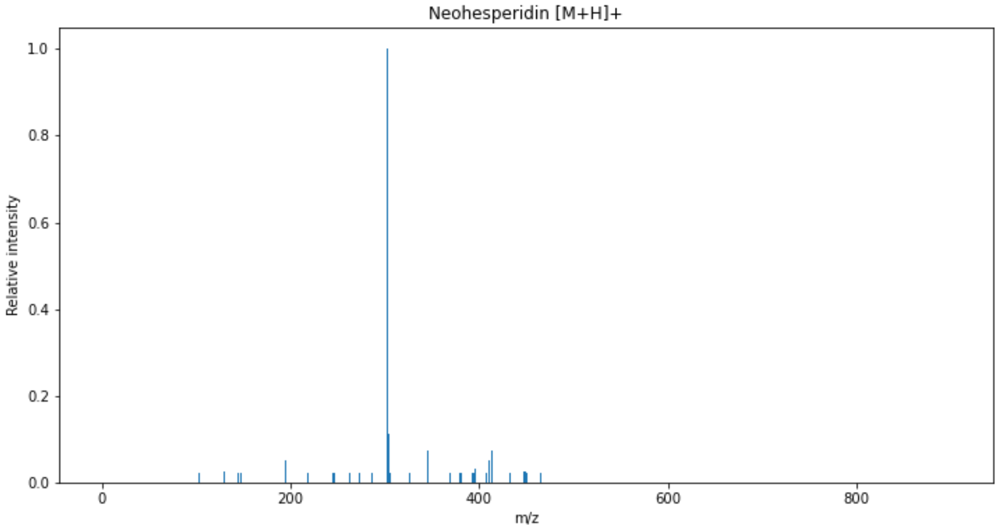

m/z **(intensity):** 103.0371 (18), 129.0465 (21), 129.0541 (21), 145.0455 (17), 148.0749 (19), 195.0311 (43), 219.0262 (18), 245.0471 (17), 247.0613 (17), 263.0641 (17), 273.0981 (17), 286.0919 (17), 303.0672 (100), 303.0852 (810), 303.1162 (25), 304.0862 (90), 305.0786 (19), 327.0709 (17), 345.0625 (27), 345.0742 (20), 345.0971 (61), 346.0949 (18), 346.1179 (18), 369.0833 (17), 369.0987 (17), 379.0627 (18), 381.0828 (19), 393.1042 (17), 395.0758 (18), 396.1248 (25), 408.087 (19), 411.1035 (43), 413.1038 (39), 413.133 (60), 414.1463 (17), 433.1384 (17), 447.1548 (20), 449.1306 (20), 450.1635 (17), 465.1527 (18)

21) Neohesperidin [M+Na]+

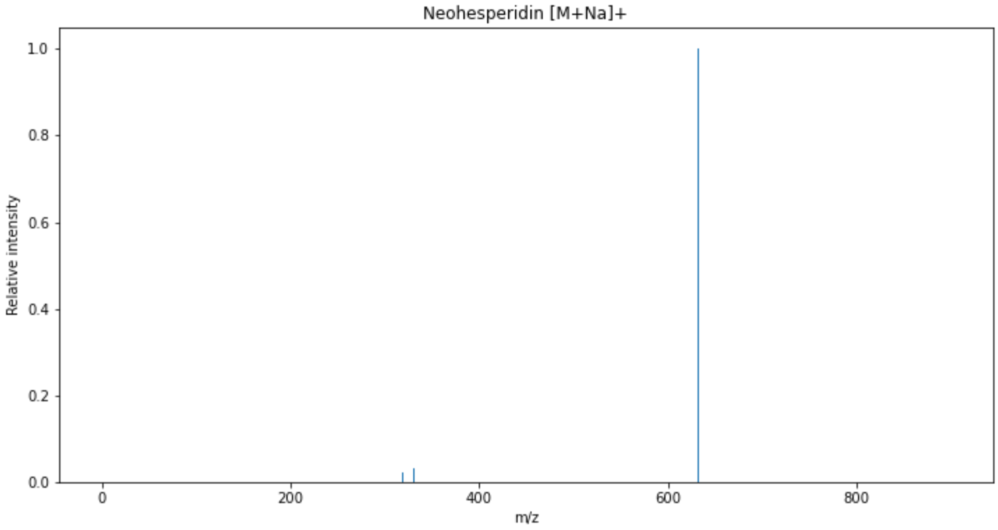

***m/z* (intensity):** 319.0157 (17), 331.0701 (24), 331.0888 (20), 633.186 (720), 634.1783 (260), 634.2012 (40), 634.2389 (26), 634.2859 (22), 634.3271 (17), 635.1648 (20), 635.2104 (26), 636.2003 (18)

22) Epimedin B [M+H]+

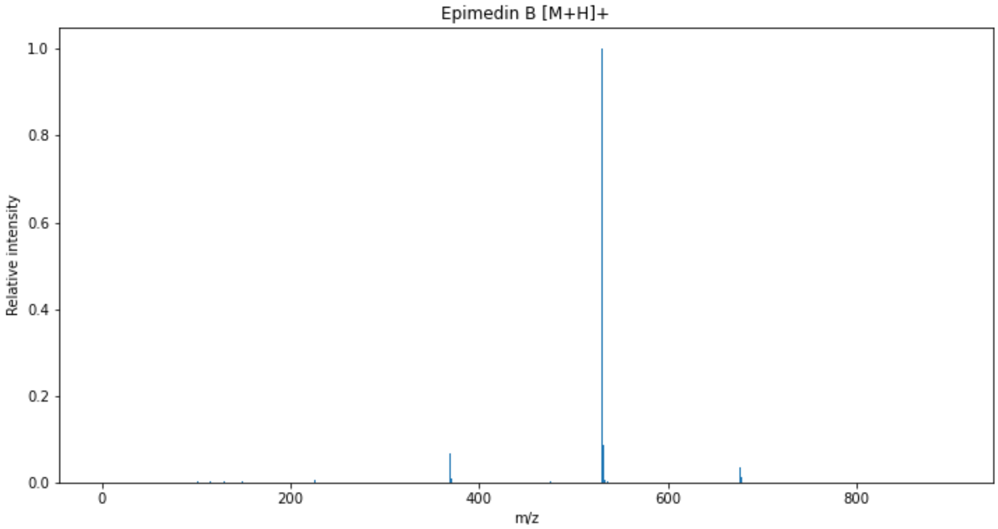

***m/z* (intensity):** 101.5627 (23), 115.0325 (21), 115.0383 (38), 119.0515 (17), 129.0459 (31), 129.052 (53), 147.0587 (18), 147.0679 (17), 149.0376 (37), 149.0444 (17), 165.0713 (17), 169.0453 (17), 171.0645 (17), 225.0718 (110), 225.095 (17), 231.083 (22), 243.073 (20), 261.0855 (18), 279.1023 (17), 280.1013 (21), 282.2707 (17), 301.0536 (19), 313.0684 (17), 314.0757 (20), 369.1047 (67), 369.1275 (950), 370.1328 (130), 475.1086 (39), 501.139 (17), 514.1802 (19), 515.1804 (17), 529.1613 (17), 530.3659 (22), 531.1854 (14000), 531.3223 (26), 532.1782 (430), 532.1913 (1200), 533.1627 (34), 533.181 (86), 533.1996 (91), 533.9574 (18), 534.1887 (17), 534.5489 (23), 536.0723 (20), 537.7137 (25), 663.2357 (19), 676.5016 (20), 677.2306 (510), 677.2568 (350), 677.6272 (20), 678.2326 (82), 678.2583 (180), 679.2748 (18), 685.3003 (17)

23) Epimedin B [M+Na]+

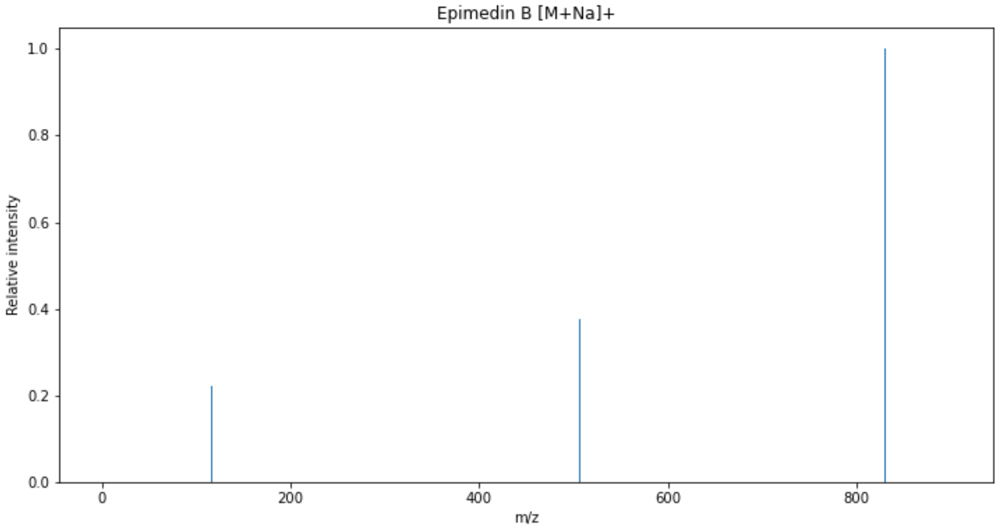

***m/z* (intensity):** 116.6667 (17), 507.2019 (29), 831.2408 (34), 831.2701 (77), 831.3323 (17), 832.2758 (20), 832.3024 (17), 833.2628 (18)

24) Icariin [M+H]+

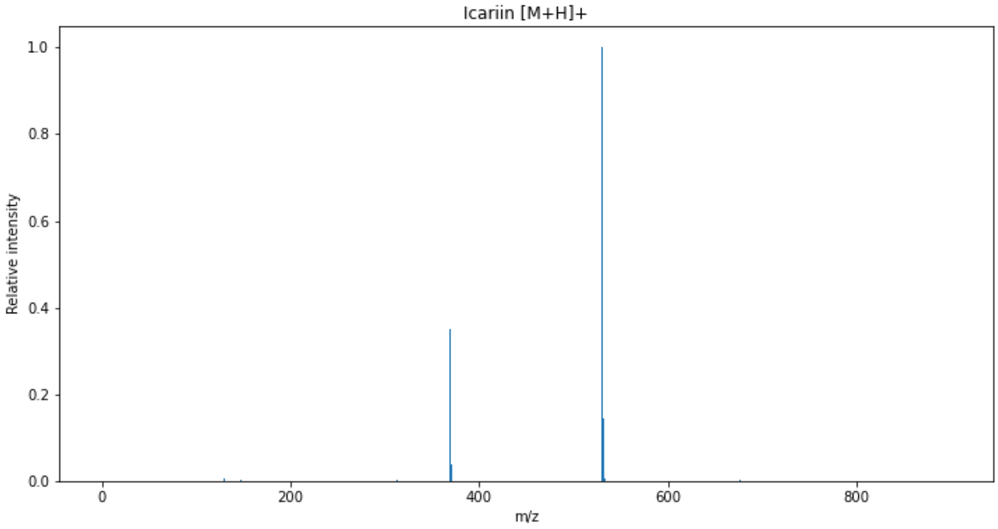

***m/z* (intensity):** 129.0522 (140), 129.0615 (17), 130.0588 (22), 147.0546 (20), 147.0594 (54), 147.067 (17), 168.3089 (17), 288.985 (20), 301.0536 (19), 301.0678 (17), 313.0562 (18), 313.071 (73), 333.0801 (17), 350.7834 (17), 358.0813 (19), 358.5694 (18), 368.452 (25), 369.0753 (38), 369.1335 (9100), 369.1896 (21), 369.236 (17), 369.3072 (20), 370.1333 (990), 370.388 (17), 371.1419 (75), 371.1599 (58), 372.1198 (18), 394.1327 (17), 417.3533 (17), 426.3467 (29), 505.1425 (17), 515.1815 (17), 516.1874 (17), 531.0781 (23), 531.122 (19), 531.1879 (26000), 531.2593 (21), 531.3394 (17), 531.6328 (19), 531.6692 (21), 532.1422 (18), 532.1912 (3800), 532.2947 (17), 533.1659 (17), 533.1906 (170), 533.2213 (18), 533.3348 (30), 533.3652 (34), 533.4122 (23), 535.0706 (18), 555.2097 (26), 557.3802 (19), 609.1918 (17), 659.2343 (17), 676.8929 (17), 677.2364 (56), 677.2547 (35), 677.2825 (17), 678.2223 (17), 679.2697 (17), 680.2429 (17)

25) Baohuoside [M+Na]+

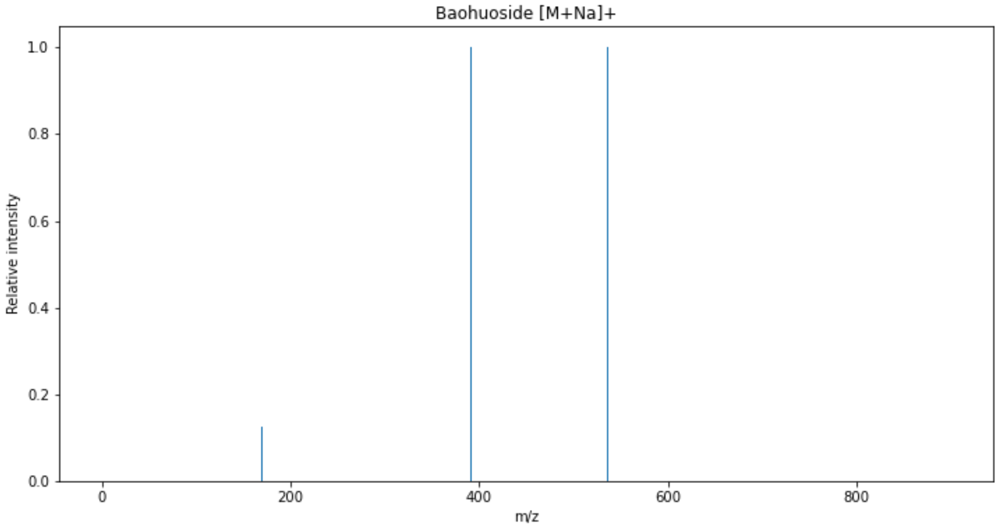

***m/z* (intensity):** 169.0526 (38), 391.113 (300), 391.1294 (77), 392.0984 (35), 392.1152 (56), 392.1286 (20), 537.176 (300), 537.2057 (36), 538.1472 (18), 538.1647 (20), 538.179 (17), 538.2047 (22), 539.1741 (17), 540.1938 (17)

S2. MS/MS of Peaks A to J in *C. unshiu* at CE 20 eV

1) Peak A

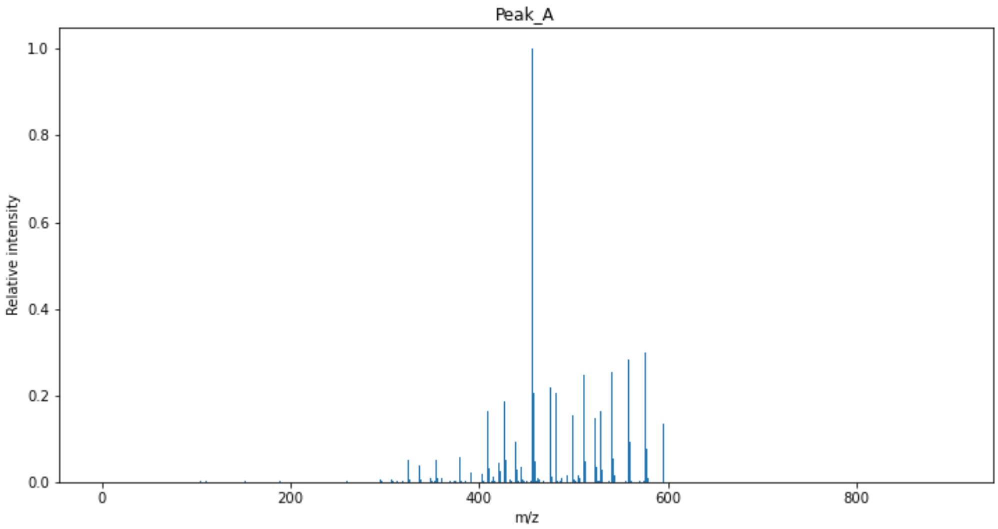

***m/z* (intensity):** 100.1788 (19), 325.0705 (880), 337.0642 (630), 338.0654 (120), 349.0612 (140), 355.0789 (880), 355.0981 (260), 356.0869 (160), 361.0793 (180), 379.0657 (410), 379.0834 (1000), 379.0988 (340), 380.0718 (170), 380.0936 (280), 391.0786 (380), 391.0925 (200), 392.0789 (120), 403.0758 (340), 409.0912 (2800), 409.1089 (740), 409.2377 (120), 410.0792 (110), 410.0998 (550), 415.12 (210), 421.0857 (780), 421.1174 (260), 422.1007 (460), 427.0787 (360), 427.103 (3200), 428.1031 (890), 433.0671 (130), 433.0995 (120), 439.1001 (1600), 439.1151 (690), 440.0891 (260), 440.1103 (490), 445.0879 (220), 445.1129 (590), 446.1161 (120), 457.1139 (17000), 458.0994 (960), 458.1176 (3500), 459.1222 (820), 463.0983 (180), 475.1229 (3700), 476.1292 (650), 477.1288 (200), 481.1122 (3500), 482.114 (700), 487.1042 (160), 493.105 (270), 499.0949 (290), 499.1241 (2600), 500.1018 (110), 500.1224 (510), 500.1497 (160), 505.1089 (290), 505.128 (140), 506.1153 (190), 511.0976 (470), 511.1238 (4200), 511.1418 (1000), 512.1328 (800), 513.1406 (200), 523.1169 (2500), 523.1409 (940), 524.1216 (620), 524.1436 (170), 529.1332 (2800), 530.1127 (140), 530.1389 (470), 531.1313 (200), 541.1317 (4300), 542.0981 (180), 542.1442 (950), 543.1369 (290), 559.1454 (4800), 560.1457 (1600), 577.1566 (5100), 578.1582 (1300), 578.1857 (320), 579.1609 (160), 595.1716 (2300), 596.1677 (680), 597.1866 (190)

2) Peak B

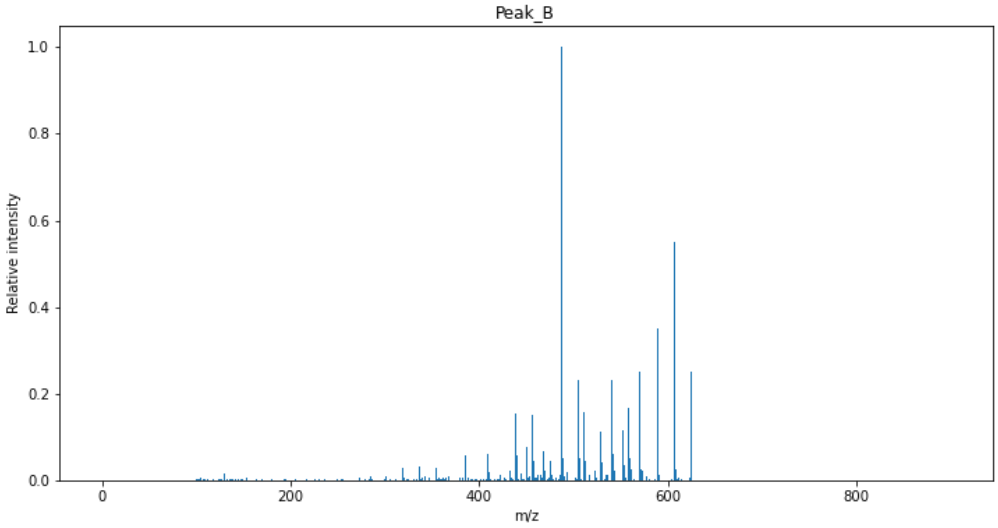

***m/z* (intensity):** 100.7547 (28), 319.0815 (180), 337.0713 (190), 355.0685 (140), 355.083 (170), 385.0979 (340), 386.0891 (150), 409.0812 (370), 409.0983 (240), 410.1006 (110), 433.0823 (140), 439.1011 (920), 440.0901 (120), 440.1105 (350), 451.1006 (460), 457.0984 (230), 457.1149 (910), 458.122 (280), 469.1015 (240), 469.1223 (400), 470.1095 (140), 475.1264 (280), 487.1044 (1400), 487.1252 (6000), 488.1193 (1000), 488.1371 (550), 488.1538 (170), 489.1244 (310), 493.1088 (110), 505.1312 (1400), 505.1496 (430), 506.128 (300), 506.1514 (240), 511.1054 (230), 511.1274 (940), 512.1273 (280), 523.1292 (140), 529.122 (530), 529.1488 (670), 530.1417 (260), 541.1073 (310), 541.1342 (1400), 542.1175 (280), 542.1425 (360), 543.1359 (130), 553.1343 (700), 554.1394 (220), 559.1437 (1000), 560.1427 (300), 561.1434 (150), 571.1274 (690), 571.1453 (1500), 572.1409 (150), 573.1626 (140), 589.1558 (2100), 590.1509 (530), 590.1833 (160), 607.1658 (3300), 608.1688 (1200), 609.1689 (160), 625.1709 (1500), 625.2049 (210), 626.2004 (280), 626.3266 (160), 627.1772 (170)

3) Peak C

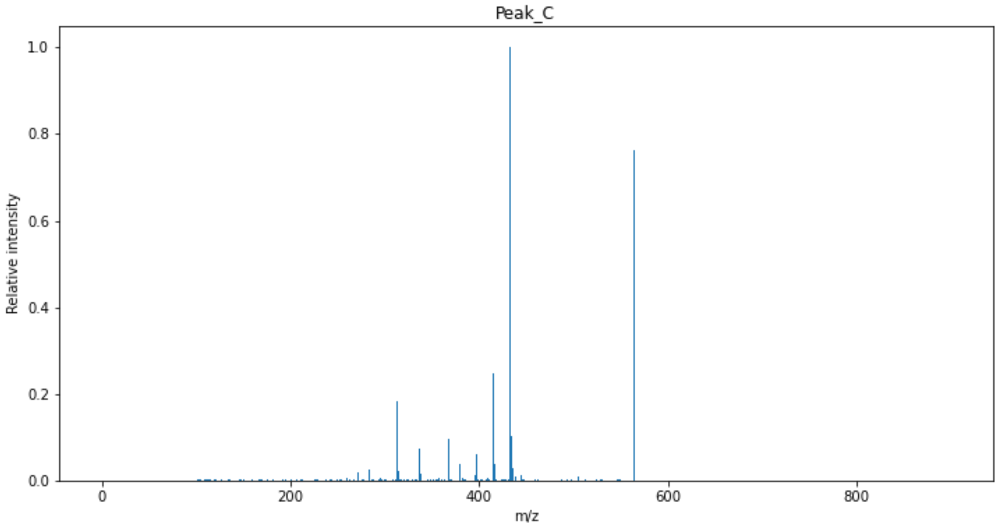

***m/z* (intensity):** 101.1898 (21), 271.0521 (190), 283.052 (190), 283.0675 (240), 313.0714 (1700), 314.0742 (200), 337.0635 (300), 337.076 (690), 338.0745 (150), 367.0712 (910), 367.0876 (510), 368.0926 (280), 379.0822 (370), 396.2323 (110), 397.0828 (580), 397.0997 (460), 398.0994 (120), 415.0881 (620), 415.1055 (2300), 416.0976 (350), 416.1225 (190), 417.1149 (130), 433.0713 (170), 433.1139 (9300), 434.0981 (450), 434.1107 (950), 434.126 (850), 435.1086 (280), 435.1293 (150), 445.103 (110), 445.1241 (130), 565.1116 (170), 565.1551 (7100), 566.1575 (2200), 567.1565 (500), 567.188 (240)

4) Peak D

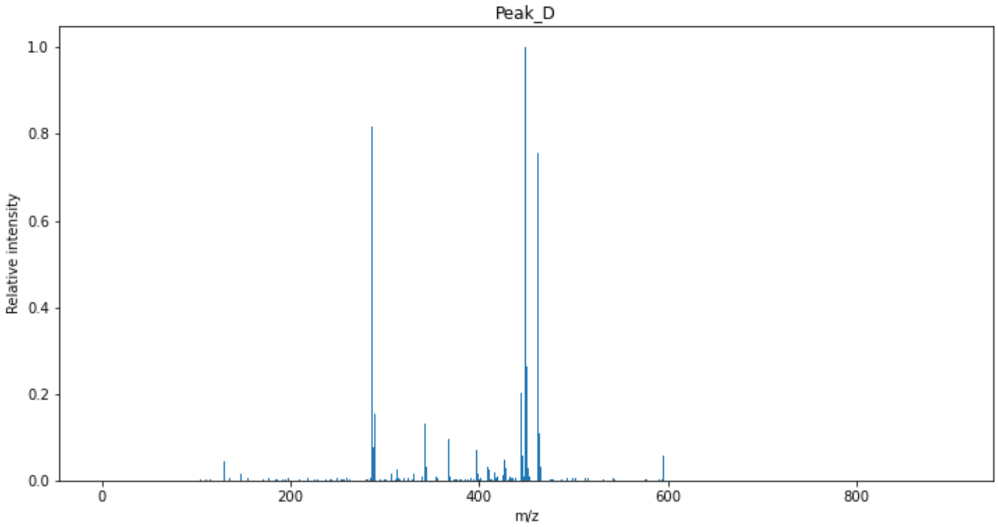

***m/z* (intensity):** 104.1129 (19), 129.0588 (220), 287.057 (4000), 288.0616 (380), 289.0705 (750), 313.0738 (120), 343.077 (650), 343.0922 (480), 344.0915 (150), 367.0876 (480), 397.0829 (320), 397.1024 (350), 409.0881 (160), 410.0893 (120), 427.0948 (230), 428.1056 (140), 445.1033 (610), 445.1204 (1000), 446.1159 (280), 449.1086 (4900), 450.1134 (1300), 451.1157 (230), 452.8753 (140), 463.1228 (3700), 464.1238 (540), 465.1205 (160), 595.1533 (280), 596.1732 (150), 596.2893 (120)

5) Peak E

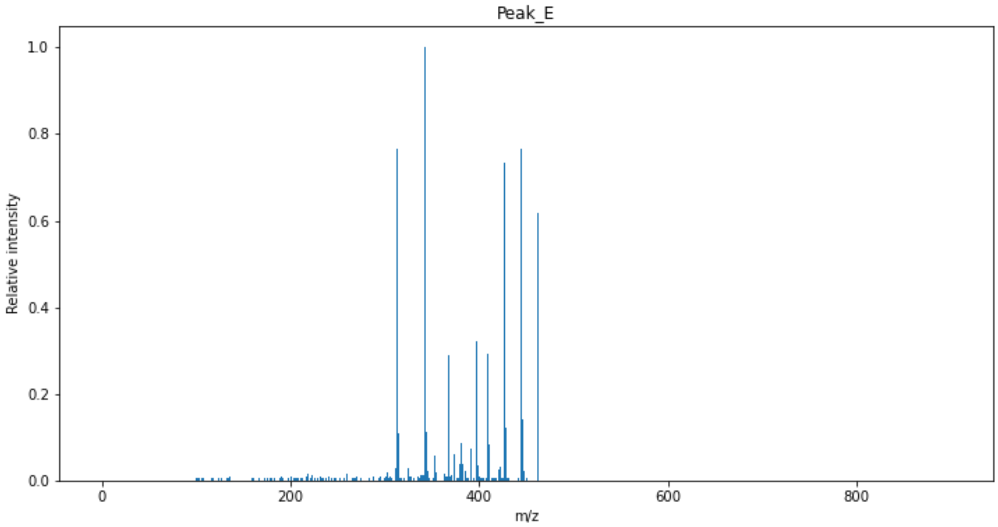

***m/z* (intensity):** 100.7793 (18), 313.0745 (2600), 314.075 (370), 343.0807 (3400), 344.0839 (380), 344.103 (110), 353.1091 (200), 367.0728 (960), 367.0874 (980), 367.1884 (130), 368.0802 (380), 368.0948 (250), 373.0882 (210), 379.0732 (130), 381.0946 (300), 382.0994 (130), 391.0809 (250), 397.0795 (570), 397.0945 (1100), 397.1139 (220), 398.0945 (370), 398.111 (160), 399.0912 (120), 409.0906 (1000), 410.089 (290), 423.1003 (110), 427.1048 (2500), 428.0903 (390), 428.1137 (420), 445.0949 (740), 445.1151 (2600), 446.0934 (190), 446.1252 (480), 463.1082 (550), 463.125 (2100), 464.1242 (550), 465.1158 (130), 465.1342 (320)

6) Peak F

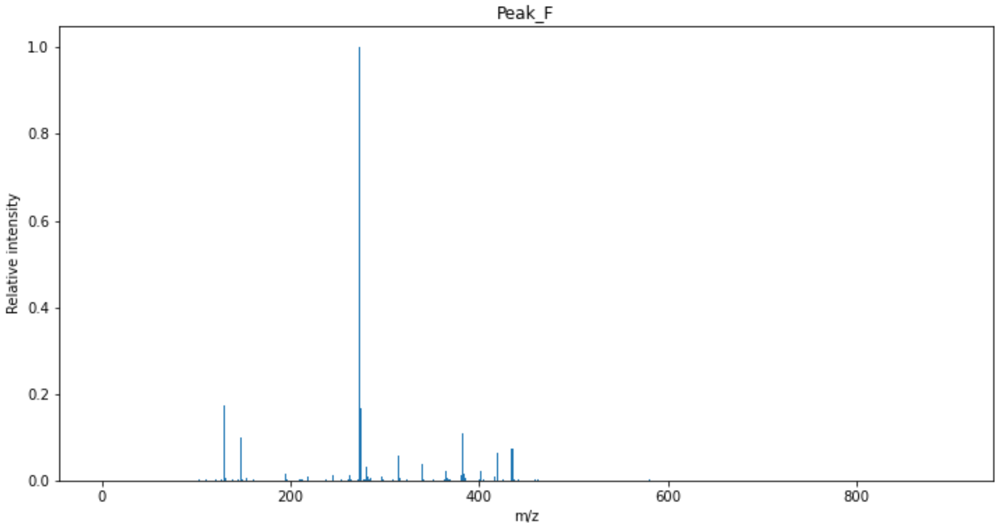

***m/z* (intensity):** 103.044 (35), 129.0577 (3300), 130.0627 (230), 147.0694 (1900), 153.019 (150), 195.0235 (120), 195.032 (300), 219.03 (170), 245.0396 (240), 263.0513 (110), 263.0612 (230), 273.0793 (19000), 274.0828 (3200), 275.0755 (110), 275.0843 (280), 281.0674 (630), 281.0785 (130), 282.0675 (200), 285.0712 (140), 297.0698 (160), 315.084 (1100), 315.1005 (360), 316.0835 (150), 316.0974 (140), 339.0578 (120), 339.0817 (750), 339.1001 (420), 340.0982 (120), 365.1045 (410), 365.1182 (240), 381.101 (250), 383.1124 (2100), 384.1006 (120), 384.1245 (300), 385.1235 (150), 401.1158 (400), 401.1298 (360), 402.1364 (240), 417.1146 (190), 419.1082 (130), 419.1347 (1200), 420.1325 (120), 435.1061 (200), 435.1305 (1400), 436.1257 (170), 436.1432 (190), 583.1465 (840), 583.17 (1300), 584.147 (150), 584.1772 (260)

7) Peak G

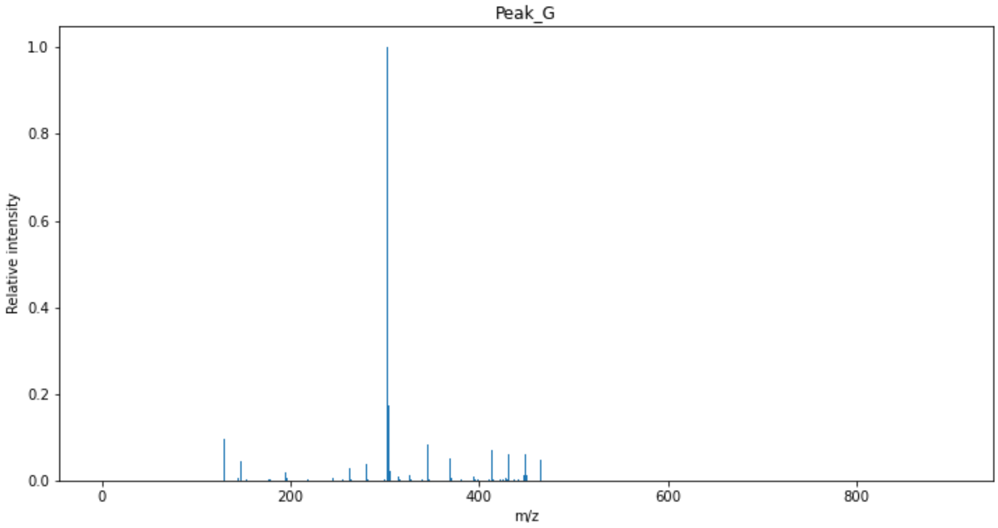

***m/z* (intensity):** 101.842 (34), 129.0577 (3900), 130.0578 (160), 145.0553 (200), 147.0685 (1800), 148.076 (150), 177.0614 (120), 179.0359 (110), 195.0303 (800), 196.0421 (250), 219.03 (160), 245.0387 (110), 245.0508 (250), 263.0594 (1200), 281.0668 (1500), 281.0818 (340), 282.0624 (120), 303.0347 (110), 303.0894 (40000), 304.0928 (6900), 305.0954 (890), 315.0729 (150), 315.09 (370), 327.0904 (520), 345.0785 (490), 345.0981 (3300), 346.0882 (280), 346.1021 (480), 369.0974 (2100), 370.1053 (300), 381.1067 (120), 395.0994 (210), 395.1111 (320), 395.125 (350), 411.1048 (140), 413.1112 (1500), 413.1264 (2800), 414.1001 (120), 414.1127 (430), 414.1287 (810), 414.1455 (200), 422.0888 (110), 429.1157 (240), 430.1168 (130), 431.1337 (2400), 432.1295 (330), 432.1505 (320), 447.1287 (490), 448.1304 (140), 449.143 (2500), 450.1299 (160), 450.1469 (490), 451.1435 (110), 465.1155 (200), 465.1401 (1900), 466.1342 (320), 466.1519 (190)

8) Peak H

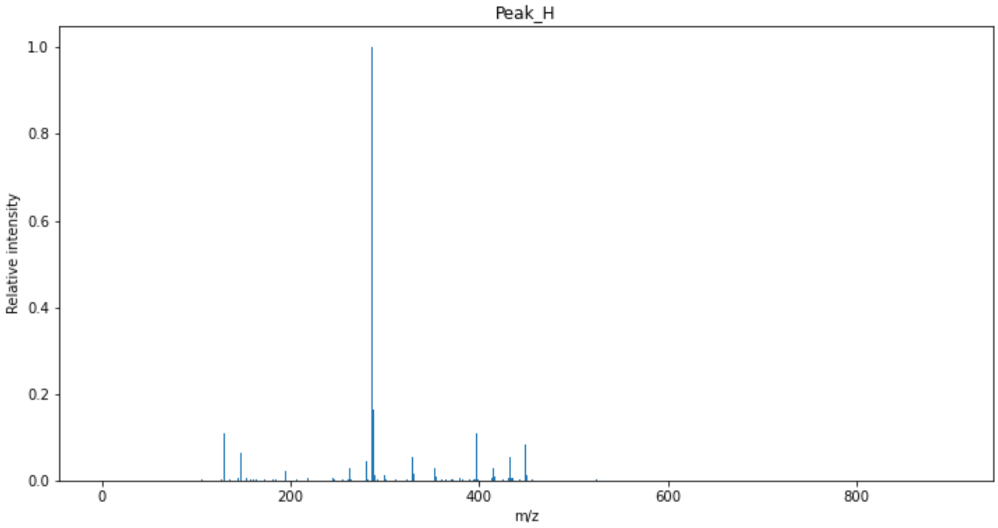

***m/z* (intensity):** 101.2659 (18), 129.0581 (2200), 145.0517 (110), 147.0684 (1300), 148.0697 (120), 153.0119 (150), 153.0253 (150), 195.0235 (140), 195.0343 (460), 195.0446 (110), 219.0343 (110), 245.0454 (160), 263.0515 (540), 263.0638 (610), 281.0694 (880), 287.0943 (20000), 288.0968 (3300), 289.0912 (130), 289.1026 (230), 299.0872 (270), 329.0934 (300), 329.1051 (1100), 329.1186 (220), 330.1063 (300), 353.0848 (140), 353.1056 (580), 354.1063 (180), 379.11 (160), 379.1292 (120), 397.1264 (2200), 398.1169 (150), 398.1343 (480), 413.127 (130), 415.1312 (560), 415.1521 (600), 416.1478 (170), 433.129 (190), 433.1505 (1100), 434.1417 (140), 435.1646 (110), 449.1433 (1700), 450.1496 (240)

3. Mirror plots between Peaks A to J in *C. unshiu* and annotated compounds from GNPS library

1) Peak A and Vicenin II

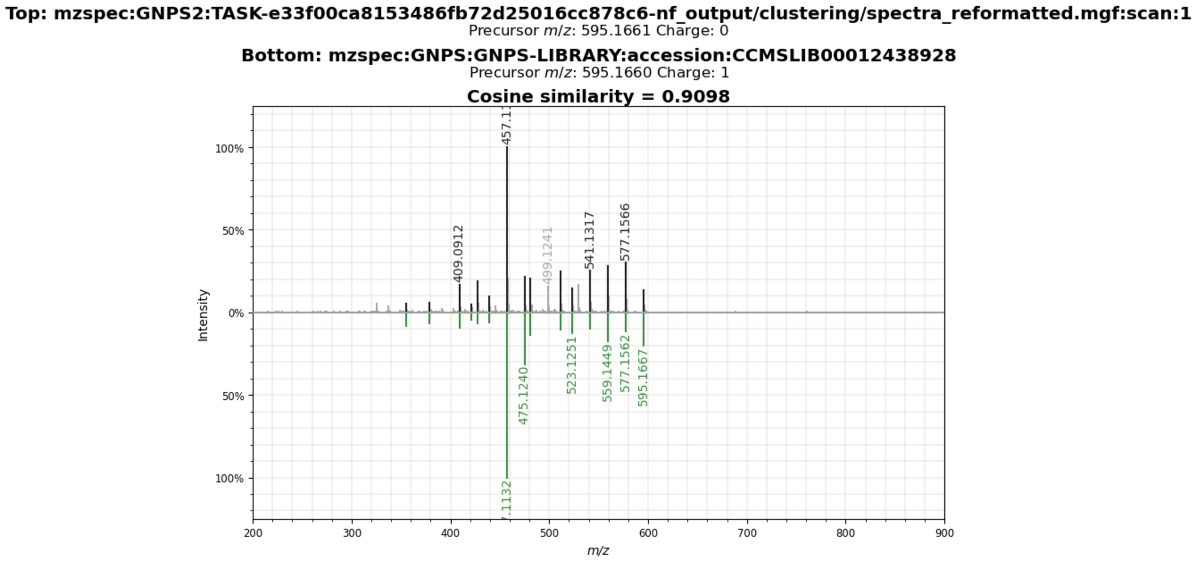

2) Peak C and Isovitexin and isovitexin 2``-O-arabinoside

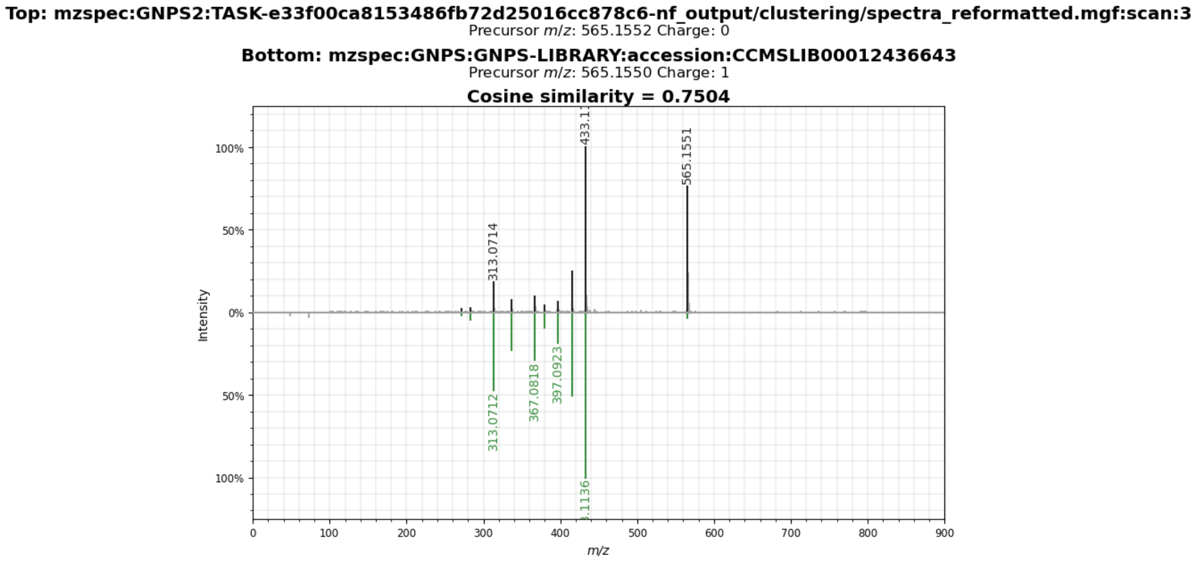

3) Peak D and nicotiflorine

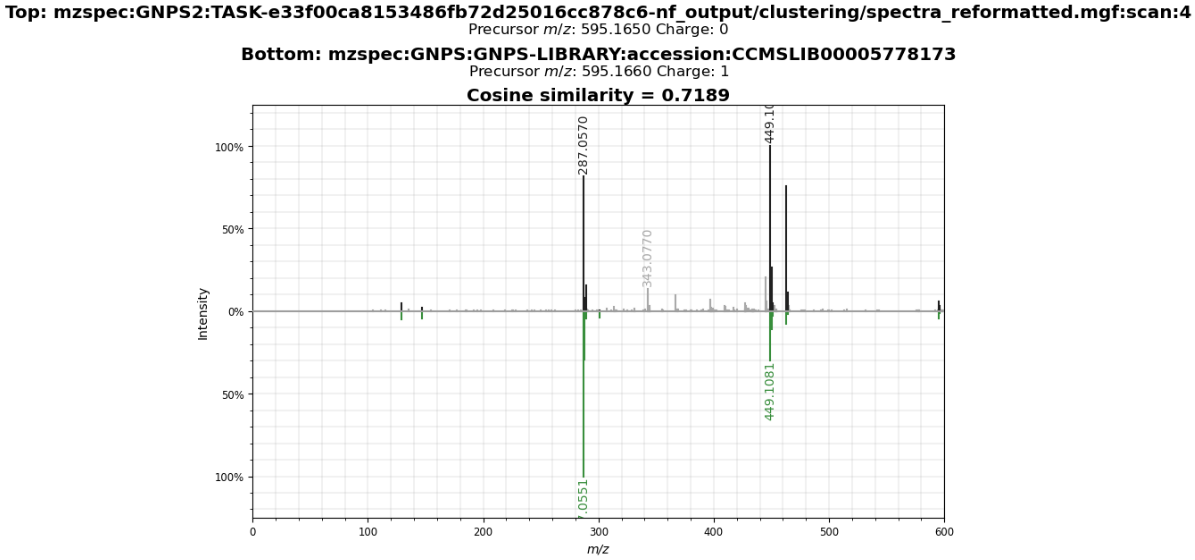

4) Peak F and naringin

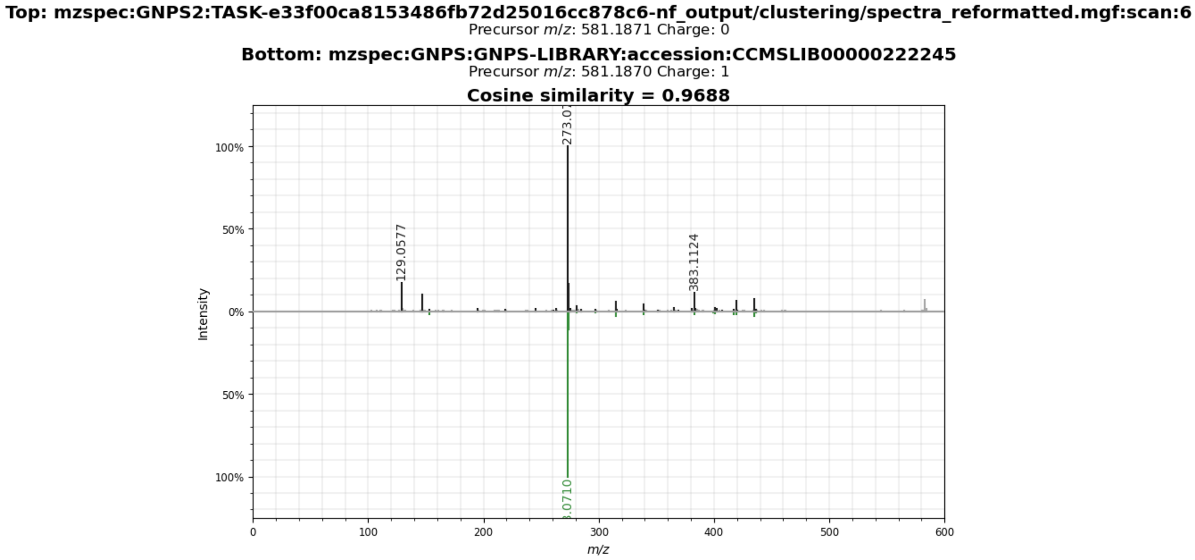

5) Peak G and hesperidin

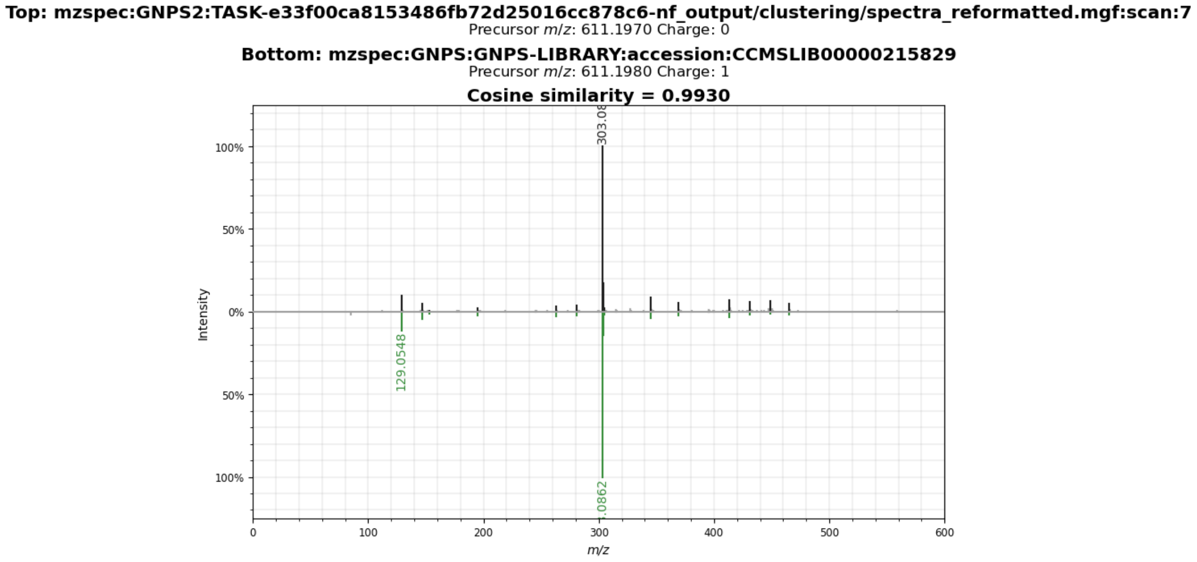

6) Peak H and poncirin

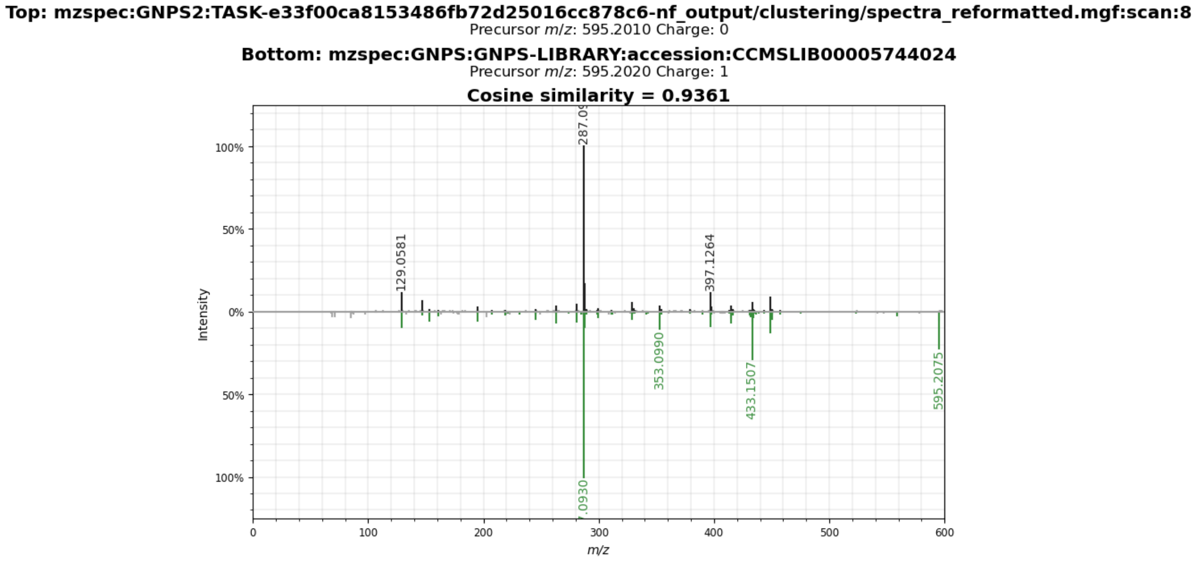

S4. Altered mass spectra of isovitexin (3)

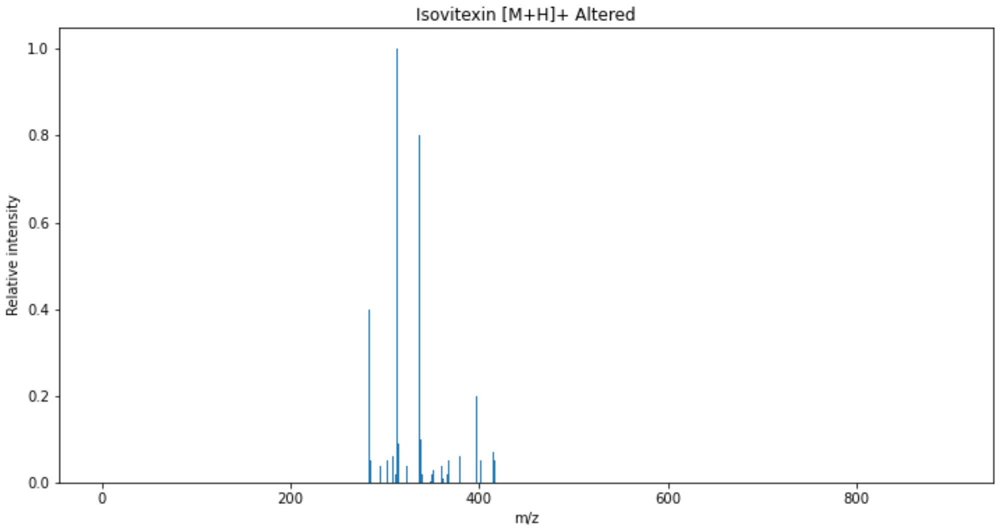

***m/z* (intensity):** 283.0596 (4000), 284.063 (700), 285.0671 (500), 295.0668 (400), 302.0304 (500), 309.0574 (600), 311.0542 (200), 313.067 (10000), 314.0713 (900), 323.0942 (70), 324.0988 (400), 337.0633 (8000), 338.0642 (1000), 339.086 (200), 340.8889 (100), 349.0605 (30), 350.0659 (200), 351.0837 (300), 361.0766 (400), 362.079 (100), 366.632 (200), 367.0737 (500), 368.0805 (80), 379.0758 (500), 380.0903 (600), 397.0922 (2000), 398.0898 (300), 402.5657 (500), 415.1033 (700), 416.1013 (500)
